# Growth Rate Variation in Brown Treesnakes (*Boiga irregularis*): An Invasive Species of Conservation Concern

**DOI:** 10.1002/ece3.71695

**Published:** 2025-07-14

**Authors:** Björn Lardner, Brian S. Cade, Julie A. Savidge, Gordon H. Rodda, Robert N. Reed, Amy A. Yackel Adams

**Affiliations:** ^1^ Department of Fish, Wildlife, and Conservation Biology Colorado State University Fort Collins Colorado USA; ^2^ U.S. Geological Survey, Fort Collins Science Center Fort Collins Colorado USA

**Keywords:** age, Gompertz growth function, quantile regression, size, snake, von Bertalanffy

## Abstract

Somatic growth rate is a fundamental trait that influences metabolism, lifespan and reproductive maturity and is critical for understanding population dynamics and informing management actions. Brown Treesnakes ( *Boiga irregularis* ) introduced to Guam are highly invasive and can reproduce year‐round without discrete cohorts. We compared snake size trajectories described by the conventionally used von Bertalanffy growth function versus the Gompertz model. Using quantile regression with a regularized effect for individual snakes we modeled growth rates of 270 marked, wild snakes as a function of size. The Gompertz model explained more of the variation in growth and rendered more realistic predictions of asymptotic sizes than did the von Bertalanffy model. With the Gompertz model, growth rates were 1.05–1.16× faster in males than in females. Females reached asymptotic sizes at shorter snout‐vent lengths than males. Growth rate was positively correlated with amount of precipitation, and modeling wet‐dry seasonality on Guam as a sinusoidal function identified a growth peak in September—October. Effects of seasonality and precipitation, however, were minor compared to individual and sex related differences in size‐adjusted growth rates. We estimated that the 50th (and 5th, 95th) growth‐rate percentile males in our study population become sexually mature at an age of 33 (∞, 15) months, while females mature at 41 (∞, 18) months, where ∞ indicates that the slowest growing snakes never reach maturity. However, 50% of the snakes mature at a size below the median, and age at maturity may be as low as 10.4 (males) and 13.7 (females) months for average‐sized hatchlings that grow fast. Our results have implications for the timing of management options for this species and our approach can be broadly applied to animals where repeated growth data are obtained and age is unknown.

## Graphical abstract

Based on a multi‐year mark‐recapture data set obtained from a population of the invasive Brown Treesnake ( *Boiga irregularis* ) on Guam, we use quantile regression and a Gompertz model to analyze how their growth varied by sex, between individuals within a sex, and as a function of annual rainfall patterns. Our results have implications for the timing of management options for this species and our approach can be broadly applied to animals where repeated growth data are obtained and age is unknown.

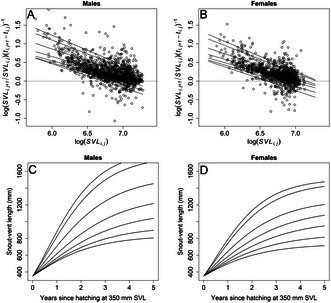

## 1 Introduction

The rate of somatic growth is a key life‐history trait in all organisms (Arendt 1997). Besides being important for management and conservation of threatened taxa (Stanford and King 2004; Rose et al. 2022) it can have profound implications for control of invasive species (Sakai et al. 2001; Nolan and Britton 2019). Management actions can be guided by characterizing variation in growth among individuals within a population, among populations (sites), temporal variation, and understanding what governs such variation (Connette et al. 2015). This applies to the invasive Brown Treesnake ( *Boiga irregularis* ), a nocturnal and cryptic predator that has had dramatic effects on the ecosystem of Guam since its accidental introduction in the late 1940s (Savidge 1987; Fritts and Rodda 1998; Rodda and Savidge 2007; Rogers et al. 2012, 2017).

Two of the most important methods for controlling Brown Treesnakes on Guam rely on live mouse (as attractants in snake traps) or dead mouse (as vector for a toxicant, placed in a bait station) lures (Nafus et al. 2022). However, Brown Treesnakes' attraction to these lures is highly size‐dependent (Tyrrell et al. 2009; Lardner et al. 2013; Klug et al. 2021). Knowing the variation in time it will take individual snakes to transition from one state (e.g., a small size at which a dead‐mouse bait is not attractive to them) to another state (e.g., larger size at sexual maturity) therefore has implications for planning and cost–benefit optimization of control efforts, which are impractical to apply continuously. For example, the allowable hiatus between management treatments must be short enough to preclude the fastest‐growing small females from reaching egg‐laying size, and long enough to have a high probability that the slowest growing individuals attain a size large enough to take the baits.

Brown Treesnakes on Guam may reproduce at any time of the year (Savidge et al. 2007) and rarely exhibit distinct age or size cohorts. We do not often encounter hatchlings (ca. 350 mm snout‐vent length (SVL)), but usually capture and mark Brown Treesnakes at a somewhat larger size (> 500 mm SVL). Assuming that growth rates are variable, these circumstances mean we rarely know the age of snakes with sufficient precision for age‐based analyses of growth. Size‐based models can help overcome this problem. A focus on size‐specific growth rates also makes sense for animals (like ectotherms) for which important life‐history shifts in ecology and sexual maturity are presumably more dependent on size than on age (Sohn and Crews 1977; Kirkpatrick 1984; Armstrong and Brooks 2013).

Different forms of the von Bertalanffy growth function have commonly been applied to reptiles (e.g., van Devender 1978; Shine and Charnov 1992; Stanford and King 2004; Rose and Todd 2020) including Brown Treesnakes (Nafus et al. 2022), but other functions may offer a better fit for more complex growth patterns (Dunham 1978; Schoener and Schoener 1978). Indeed, of many nonlinear growth functions that approach an asymptote, the von Bertalanffy has a restrictive requirement that the greatest rate of change in the growth function must occur at the smallest measured size (Goshu and Koya 2013) which can hinder acceptable function fit at larger sizes. Other nonlinear functional forms that approach an asymptote such as the Gompertz do not have this restriction (Goshu and Koya 2013) and have been found to provide better growth functions for some taxa (Troynikov et al. 1998). While data on known‐age animals are helpful, such knowledge is not necessary to infer a size‐by‐age relationship (van Devender 1978). Usually, one function is fitted to data from a population to describe the general growth pattern across all individuals (e.g., Plummer 1985; Maritz and Alexander 2011). Confidence intervals around that function have been used to infer possible shapes of the true size‐age relationship (e.g., Blouin‐Demers et al. 2002). Bayesian (Eaton and Link 2011; Sigourney et al. 2012) or mixed‐effects (Hart and Chute 2009) approaches to growth modeling can be used to tease out individual variation. Building on such a framework, Armstrong and Brooks (2013) explicitly addressed individual variation in growth parameters based on size (not age) in turtles, while simultaneously allowing for sex differences in resource allocation after maturation.

For our study population of Brown Treesnakes, we wanted to estimate population‐level parameters that would account for heterogenous variation in individual snake growth rates as well as differences between sexes to improve management interventions such as suppression and eradication. Failure to account for accurate growth variability in suppression modeling efforts could impede successful interventions. We considered von Bertalanffy and Gompertz growth models and evaluated which model offered a superior fit to data. Building on the model that performed best, we then assessed the degree to which environmental factors such as wet‐dry seasonality or variation in precipitation explain intraspecific variation in growth. We used quantile regression (see Cade and Noon 2003 for a primer on this regression approach) to characterize among‐snake variation in growth rates by estimating different growth rates for slower to faster growing snakes, coupled with a random‐effect‐like shrinkage estimate for among individual snake variation. Quantile regression previously has been used to model variation in growth for fish populations (Cade et al. 2008, 2011) as well as estimating the effects of habitat type, sex, and seasonal variation through a range of snake body length quantiles (Siers et al. 2017). Quantile regression allows us to estimate growth relationships at multiple quantiles (slower to faster growth rates at lower to higher quantiles) of the growth rate distribution rather than just the mean as in conventional regression models. The shrinkage estimates reduce differences among individual snakes, with greater reduction for snakes with smaller sample sizes and more extreme values, improving interpretation and generalization to the population of snakes (Tibshirani 1996). Assuming a particular (mean) size at hatching, we could then estimate growth‐by‐age functions for slow/average/fast growing males and females. More complex models estimated with additional environmental covariates allowed us to also estimate the joint effect of precipitation and wet‐dry seasonality.

## 2 Materials and Methods

### 2.1 Study Population and Survey Methods

We used mark‐recapture data to estimate snake growth in a Brown Treesnake population in a 5‐ha plot located on NW Guam (13.639° N, 144.862° E, datum WGS 84). In early 2004, the plot was enclosed by a fence that prevents both emigration and immigration of snakes but not their prey of geckos, skinks, and rodents. It has since been surveyed periodically, and we here use input data spanning from 10 May 2004 to 18 July 2013. Snakes were located by visual searches at night, by mouse‐lure trapping using commercial minnow traps fitted with wire mesh entrance flaps, after they took radio‐transmittered bait, and by incidental encounters (Rodda et al. 2007; Tyrrell et al. 2009; Christy et al. 2010; Lardner et al. 2013). PIT tags (passive integrated transponders) and ventral scale clips allowed individual identification. All field work was conducted in accordance with Colorado State University IACUC Protocols.

### 2.2 Data and Analyses

Our data consisted of multiple snout‐vent length (SVL) measures for each snake at irregular intervals, obtained by stretching the snake along a measuring tape by highly trained biologists. Such measures are associated with a measurement error (Madsen and Shine 2001; Blouin‐Demers 2003; Luiselli 2005). We only used measurements from each individual snake that were ≥ 20 days apart to minimize the influence of measurement errors, especially those resulting in negative growth rates between successive measurements.

Our primary focus was on growth in snakes from immature to mature stages, so we limited the analysis to snakes with an initial SVL measurement ≤ 850 mm, below the size ranges at which the average male and female snake on Guam matures (90% range: male 940–1030 mm; female 910–1025 mm: Savidge et al. 2007), and with at least a 150 mm increase in SVL. We also only included snakes with ≥ 4 measurements. The above rules rendered a data set of 270 snakes (128 females, 142 males), with a median of 10 measurements (range = 4–39), and with a median of 64 days (range = 20–866 days) between successive measurements over a median time frame of 1012 days (range = 133–2398 days). Initial measurements for individuals were a median SVL of 537 mm (range = 320–842 mm) and increases in SVL for individuals were a median of 484.5 mm (range = 151–1061 mm).

### 2.3 Gompertz Model

We used a growth‐increment form of a Gompertz model to estimate growth rates of snout‐vent length (SVL) in Brown Treesnakes: SVL_*i*, *j* + 1_ = SVL_*i*, *j*_(exp(*β*_0_ + *β*_1_ × log(SVL_*i*, *j*_)))^(*t*^_*i*, *j* + 1_ − ^*t*^_*i, j*_^)^, where SVL_*i*, *j* + 1_ at time *t*_*i, j +* 1_ is a multiplicative function of SVL_*i*, *j*_ and the logarithm of SVL_*i*, *j*_ at the previous time *t*_*i, j*_ for the *i*th individual snake raised to the power of the interval between times *t*_*i, j*_ and *t*_*i*, *j* + 1_ for SVL and *t* temporally ordered by *j* for each of *I* snakes. This growth‐increment form of the Gompertz model is similar to one often used in modeling density‐dependent changes in population size (e.g., Cade et al. 2022). This was expressed as a linear quantile regression model *Q*_**y**_(τ|**X**) = **Xβ(**τ), where **y** = log(SVL_*i*, *j* + 1_/SVL_*i*, *j*_) × (*t*_*i*, *j* + 1_ − *t*_*i, j*_)^−1^ is (*n* − *I*) × 1 matrix of growth increments, **X** is (*n* − *I*) × *p* matrix of predictors including a column of 1's for an intercept and a column for log(SVL_*i*, *j*_), and **β** is *p* × 1 matrix of parameters corresponding to **X** and indexed by τ ∈ [0, 1] for the inverse of the unknown cumulative distribution function (Cade and Noon 2003; Koenker 2005). Sample size and matrix rows were *n* − *I*, where *n* is total number of snake observations and *I* is number of individual snakes, because of losing 1 observation for each of *I* individual snakes by forming ratios between temporally ordered measurements. The **X** matrix of predictors was expanded to include additional predictors to define sex of snakes and environmental covariates as required for analyses.

To account for among‐individual variation in snake growth rates and the repeated measurements of *I* individual snakes (SnakeID), we estimated a quantile regression model *Q*_*y*_(τ|(**X**, **Z**) = **Xβ**(τ) + **Zα**(τ) for τ ∈ [0, 1] that included a predictor variable **Z** that allows differences in intercepts for individual snakes of each sex, SEX_*i*_ × SnakeID_*i*_. This model allows heterogeneity in the effects of log(SVL_*i*, *j*_) and sex expressed through the quantiles where higher to lower τ reflect faster to slower growth in snakes, but variation among individuals at a quantile is reflected in changes among intercepts only. The differences in intercepts for individual snakes **α**(τ) were estimated with shrinkage using the least absolute shrinkage and selection operator (LASSO, Tibshirani 1996), providing reduced parameter and regularized estimates compared to highly parameterized fixed‐effect estimates (Koenker 2011). Thus, terms in **α**(τ) for differences in intercepts among individual snakes were centered on fixed‐effect estimates for the intercept (females) and differences in intercepts for SEX_*i*_ (males), similar to estimating random effects on intercepts. Although we had 270 individual snakes and, thus, potentially 270 intercepts and differences to estimate, the expected benefit of LASSO shrinkage is that many of those estimates will shrink to zeros. We transformed our response variable **y** = log(SVL_*i*, *j* + 1_/SVL_*i*, *j*_) × (*t*_*i*, *j* + 1_ − *t*_*i, j*_)^−1^ from daily to annual growth rates by multiplying by 365 days to provide larger values for the LASSO shrinkage to better detect effective degrees of freedom based on zero residuals.

We estimated the linear quantile regression model with LASSO shrinkage on individual snake intercepts in the R computing environment (version 4.0.2; R Core Team 2020) using the rqss() function of the quantreg package (version 5.55; Koenker 2014) for quantiles τ = {0.05, 0.10, 0.25, 0.50, 0.75, 0.90, 0.95}. Because the LASSO requires that a shrinkage parameter λ be specified for each τ, we explored across a grid of values for λ = 0.1–6.0 minimizing the Bayesian Information Criterion (BIC) to select the most parsimonious value of λ for a given model and quantile. We chose to use BIC for model selection because the log(*n*) multiplier of number of parameters *p* tends to select models with fewer parameters than Akaike Information Criterion (AIC), which uses 2 × *p*, and BIC is directly related to evidence functions (Dennis et al. 2019). Smaller values of λ imply less shrinkage and more parameters to estimate, approaching a fixed effect estimate for individual intercepts with no pooling of information across individual snakes. Larger values of λ increase shrinkage by reducing the number of intercept parameters to estimate for individual snakes, approaching a completely pooled analysis with no individual snake intercepts. Standard errors of estimates for fixed‐effect parameters were computed with the Hendricks and Koenker sandwich estimator (Koenker 2005, 2011). We considered models that pooled across both sexes, had common slopes but different intercepts between sexes (**X** includes a factor variable SEX_*i*_ with 0 if female and 1 if male), and separate slopes and intercepts between sexes (**X** also includes SEX_*i*_ and its interaction with log(SVL_*i*, *j*_). We also computed *R* ^1^ coefficients of determination for each quantile estimate that compared the proportionate reduction in variation between models that included the log(SVL_*i*, *j*_) and those without this term (Koenker and Machado 1999) to evaluate fit of the Gompertz model form. The *R* ^1^ coefficients of determination measure the proportionate reduction in absolute deviations between estimates and observations for quantile regressions from including the predictor variable, providing a similar function as *R* ^2^ coefficients of determination for mean regression. We computed standard deviations among predicted growth rates for individual male and individual female snakes by quantiles for our final model to further compare individual variation among snakes by sex.

### 2.4 von Bertalanffy Model

We also considered a growth‐increment version of the von Bertalanffy growth model, following procedures in Hart and Chute (2009) and Paragamian and Beamesderfer (2003). The growth increment form of the von Bertalanffy model was SVL_*i*, *j* + 1_ = SVL_*i*, *j*_ + (SVL_∞_ − SVL_*i*, *j*_) [1 − exp.(−*K* (*t*_*i*, *j* + 1_ − *t*_*i, j*_))], where SVL_*i*, *j* + 1_ at time *t*_*i, j* + 1_ is a function of SVL_*i*, *j*_ at the previous time *t*_*i*, *j*_ for the *i*th individual snake and a multiplicative function of the difference between SVL_*i*, *j*_ and the asymptotic size SVL_∞_, and growth coefficient *K* multiplied by the time interval *t*_*i, j* + 1_ − *t*_*i*, *j*_. We estimated this as a linear quantile regression model *Q*_**y**_(τ|**X**) = **Xβ(**τ), where **y** = (SVL_*i*, *j* + 1_ − SVL_*i, j*_) × (*t*_*i*, *j* + 1_ − *t*_*i, j*_)^−1^ is (*n* − *I*) × 1 matrix of average growth rates, **X** is (*n* − *I*) × *p* matrix of predictors including a column of 1's for an intercept and a column for the mid‐point of prior and current SVL, (SVL_*i*, *j* + 1_ + SVL_*i, j*_)/2, and **β** is *p* × 1 matrix of parameters corresponding to **X** (Paragamian and Beamesderfer 2003). Time intervals (*t*_*i*, *j* + 1_ − *t*_*i, j*_) were expressed as fractions of a year as in Gompertz models. The von Betalanffy growth coefficients were estimated as *K*(τ) = −(log(${\hat{\beta }}_{1}$(τ) + 1)) and asymptotes were estimated as SVL_∞_(τ) = −${\hat{\beta }}_{0}$(τ)/${\hat{\beta }}_{1}$(τ), where ${\hat{\beta }}_{0}$(τ) and ${\hat{\beta }}_{1}$(τ) are estimates from the linear quantile regression models. As with Gompertz growth models, we included terms for different intercepts of individual snakes by sex that were regularized with the LASSO estimator in rqss() function. Again, we selected appropriate λs for each τ by minimizing BIC across a grid of values for λ = 0.1–6.0 for a given model and quantile. We computed *R* ^1^ coefficients of determination for each quantile estimate that compared the proportionate reduction in variation between models that included the mid‐point of SVL predictor and those without this term (Koenker and Machado 1999) to assess proportion of variation explained by von Bertalanffy compared to Gompertz growth models.

### 2.5 Seasonality and Precipitation

Guam has a tropical maritime climate with average monthly night‐minimum temperature fluctuations of merely 1.4°C during a year (Casey 2021). However, there is distinct wet‐dry seasonality with July–November having a mean monthly precipitation of 295 mm and December–June a mean monthly precipitation of 120 mm (Casey 2021). Our next goal was therefore to develop models that added effects of seasonality and precipitation on growth. We obtained rainfall data from the weather station at Guam International Airport, ca 18 km from our study site, spanning from March 2003 to July 2013 from the National Oceanic and Atmospheric Administration's “NOWdata – NOAA Online Weather Data” (www.weather.gov/wrh/Climate?wfo=gum; accessed on 04 January 2022). For the sole date with missing data (27 June 2004, during Typhoon Tingting), we used the value 416 mm (see the Supporting Information for justification). Starting > 1 year prior to the first growth rate estimate, we calculated for each date the cumulative precipitation (mm rain) the previous 90 days (90RAIN). Precipitation and seasonality covariates were included in our quantile regression models with reference to the end date *t*_*i*, *j* + 1_ of growth intervals for each individual *i*th snake.

To assess if snake growth correlates to the island's distinct wet‐dry seasonality (Figure S1) we first considered models that included a sinusoidal SEASON covariate with a phase of 365 days and values oscillating between −1 and + 1, lagged between 0 and 51 weeks. This sinusoidal variable may be thought of as an idealized form of seasonality—it remains the same across the different years of our study and undulates regularly, with a 1‐year phase, according to the maxima and minima that evaluation of 52 (weekly) phase‐shifted models eventually identified. As an alternative approach that addresses the same question, we considered models that instead of SEASON included variable 90RAIN, the cumulative rainfall over the 90 preceding days. There may be delayed effects of rain caused, for example, by lagged responses in snake prey populations (Rose et al. 2018), so for each quantile (in models still containing effects of SVL and sex) we estimated 52 models for 90RAIN: one with the un‐lagged data and 51 models with the cumulative precipitation lagged one additional week for each consecutive model run. We selected models to consider further by calculating BIC values for lags by quantile and compared these to BIC for the reduced model counterparts that lacked the seasonality or rainfall variable. Because these comparisons were unlikely to provide a consistent choice across all quantiles estimated, we graphed results and considered those lags where median differences in BIC across quantiles were greatest.

To address how precipitation deviations from the regular wet‐dry seasonality affect snakes, we then investigated how 90RAIN interacts with SEASON. The latter variable was fixed to the lag(s) previously identified. We again estimated 52 models for the 90RAIN variable and its interaction with seasonality: one with the un‐lagged data and 51 models with the cumulative precipitation lagged one additional week for each consecutive model run. To some extent, variables SEASON and 90RAIN are two sides of the same coin. By fitting the 90RAIN variable after having fixed the sinusoidal SEASON variable at its previously identified optimal lag (or really: phase shift) we hoped to detect any signal in snake growth from precipitation patterns deviating from the seasonal norm—be it a more pronounced seasonal precipitation pattern than usual (i.e., excessive rain during the wet season and/or extreme drought during the dry season) or, alternatively, a less pronounced seasonal rainfall pattern than usual (i.e., an unusually dry wet season and/or an unusually wet dry season). We calculated BIC values for the lags by quantile and compared these to the BIC for the reduced model counterparts that lacked precipitation and seasonality variables. Again, we graphed BIC differences across lags and used the median difference in BIC across quantiles for each lag to select reasonable lags for further evaluation. For these, we calculated partial effects and their confidence intervals by quantile, and plotted response surfaces of partial effects when including environmental covariates. We also compared *R* ^1^ coefficients of determination to estimate the importance of adding these covariates to the model.

## 3 Results

**FIGURE 1:**
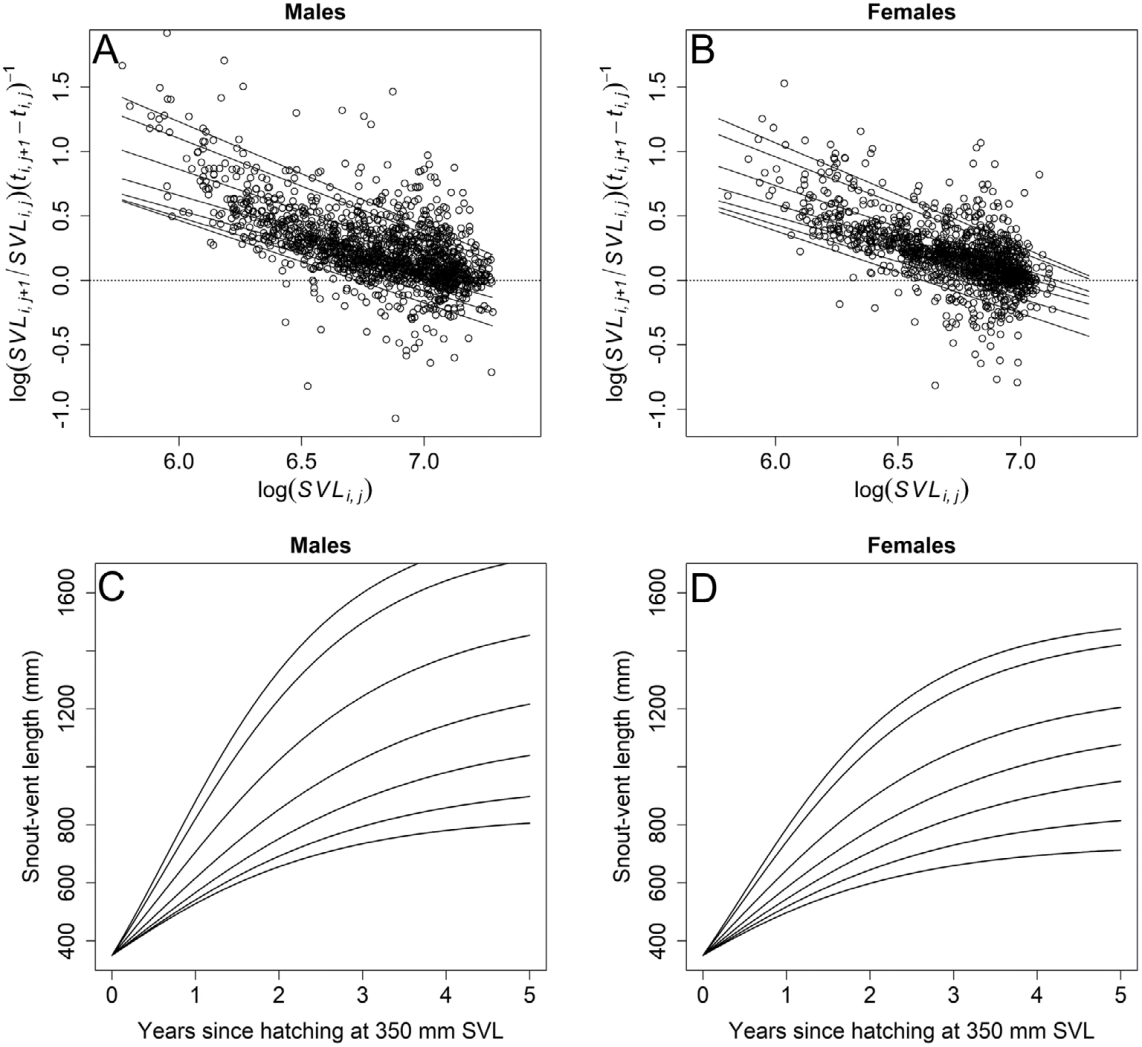
Quantile regression (τ = {0.05, 0.10, 0.25, 0.50, 0.75, 0.90, 0.95}; lower to higher lines) estimates of male (*n* = 1517 for 142 individuals) and female (*n* = 1302 for 128 individuals) Brown Treesnake growth rates as a multiplicative function of logarithm of prior snout‐vent length (SVL_*i*, *j*_) in a Gompertz growth model. Estimates were obtained from linear quantile regression models of logarithm of annual growth rates in a model with common slopes but different intercepts for males (A) and females (B) with least absolute shrinkage and selection operator (LASSO) shrinkage on intercepts for individual snakes. Panels (C, D) are the corresponding multiplicative Gompertz estimates made by starting growth at 350 mm SVL, demonstrating larger SVL and greater size variation for males compared to females. Measurements were made on Brown Treesnakes in a 5‐ha plot located in NW Guam from 10 May 2004 to 18 July 2013.

**FIGURE 2:**
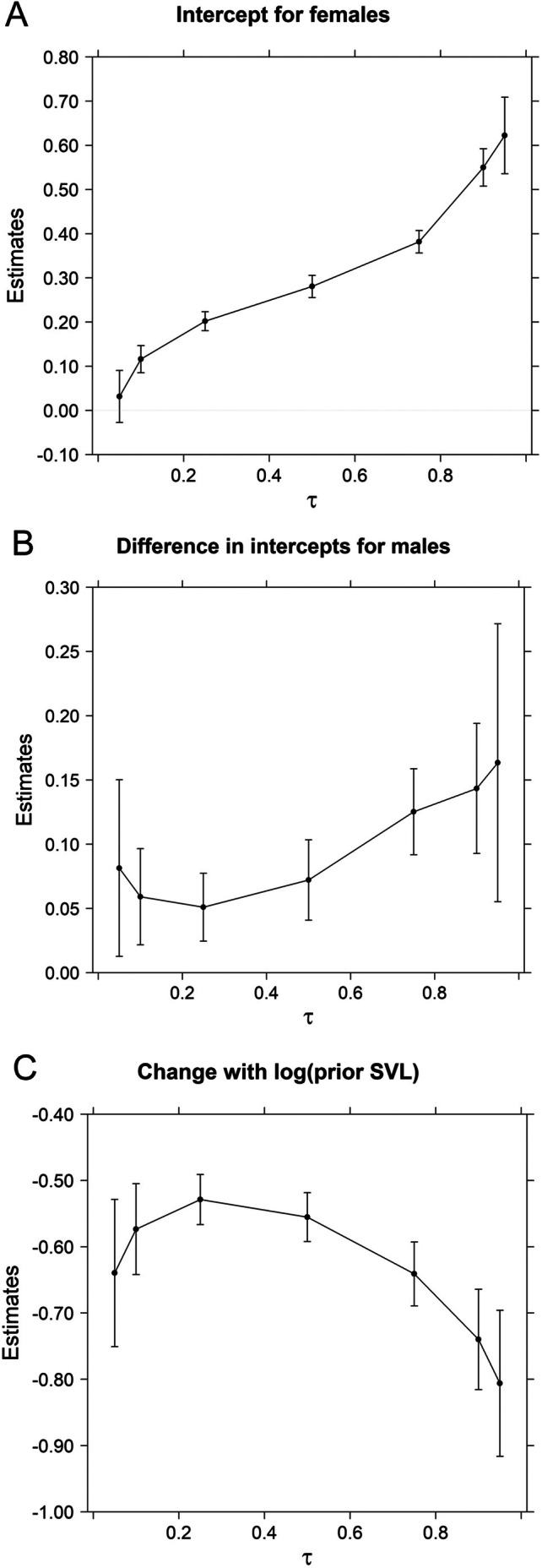
Parameter estimates and 95% confidence intervals for linear quantile regression (τ = {0.05, 0.10, 0.25, 0.50, 0.75, 0.90, 0.95}) models of female (*n* = 1302 for 128 individuals) and male (*n* = 1517 for 142 individuals) Brown Treesnake growth rates measured in a 5‐ha plot located in NW Guam from 10 May 2004 to 18 July 2013. The model is a linearized version of a Gompertz model where logarithm of annual growth rates has a common relationship with logarithm of prior snout‐vent length (SVL_*i*, *j*_) for males and females (C) but intercepts—scaled so intercept is log (700 mm) – can differ for females (A) and males (B). Linear quantile regression models included a least absolute shrinkage and selection operator (LASSO) shrinkage estimate on intercepts for individual snakes.

The Gompertz models consistently outperformed von Bertalanffy models by explaining more of the growth variation. Our Gompertz growth models with LASSO shrinkage on intercepts for individual snakes had λs ranging from 1.0 to 4.5, substantially reducing the 270 individual snake intercepts to 36, 17, 21, 31, 27, 24, and 110 nonzero values for τ = 0.05, 0.10, 0.25, 0.50, 0.75, 0.90, and 0.95, respectively. Models with separate intercepts and slopes for both sexes were not well supported as differences in slopes between males and females had *p* > 0.494 across all τ except τ = 0.25 where *p* = 0.109. Models with separate intercepts but common slopes for sexes were well supported as differences in intercepts (scaled to equal log(700 mm)) had *p* < 0.002 for all τ except τ = 0.05 where *p* = 0.020. Linear forms of this model (Figure 1A,B) had *R* ^1^ coefficients of determination of 0.131, 0.138, 0.203, 0.232, 0.231, 0.237, and 0.210 for τ = 0.05, 0.10, 0.25, 0.50, 0.75, 0.90, and 0.95, respectively. Exponentiated estimates of differences between male and female intercepts (Figure 2B) indicated males had 1.051–1.162× greater annual growth rates than females, with differences generally increasing for faster growing snakes at higher quantiles. Annual growth rates of females ranged from 1.04 for slower‐growing (τ = 0.05) to 1.86 for faster‐growing (τ = 0.95) individuals at prior SVL = 700 mm (exponentiated estimates in Figure 2A). The negative relationship of growth rates with prior SVL (SVL_*i*, *j*_) increased with increasing quantile, although there were slightly greater negative growth rates at lowest quantiles too (Figure 2C). The ratio of standard deviations of predicted quantiles for individual males compared to individual females increased from near 1 at lower quantiles (1.08, 0.73, and 1.08 for τ = 0.05, 0.10, and 0.25, respectively) to greater than 2 at most higher quantiles (2.65, 2.08, 3.60, and 1.72 for τ = 0.50, 0.75, 0.90, and 0.95, respectively), consistent with greater variation among males compared to females at higher quantile (τ ≥ 0.50) estimates when averaged across individuals (compare Figure 1A,B).

We expect by definition that 90% of the observed SVL of all snakes will be contained within an interval formed by 0.05 and 0.95 quantile estimates. If we have modeled individual variation well, we would also anticipate that there might be a similar proportion of observed SVL measurements of individual snakes contained within their 0.05 and 0.95 quantile estimates. We graphed a selection of individual males and females including those with sparse, moderate, and denser sampling over time to demonstrate estimates of quantile intervals with respect to observed SVL (Figure S2). Estimated 0.05 and 0.95 quantiles of SVL bracket large but differing proportions of individual SVL measurements, indicating that our modeling of individual snake variation was effective for most but not all individual Brown Treesnakes. We found 85% of 270 snakes had 75%–100% of their observed SVLs within estimated 0.05–0.95 quantiles (Figure S3). The other 15% of snakes had 21%–74% of their observed SVL within those quantile intervals, indicating poorer fit for a small proportion of individual snakes.

Estimating nonlinear Gompertz functions from the quantile estimates assuming a common starting SVL = 350 mm clearly indicates greater growth rates of male compared to female Brown Treesnakes and that males attain greater SVL over time (Figure 1C,D). Equilibrium growth rates [exp(−${\hat{\beta }}_{0}$(τ)/${\hat{\beta }}_{1}$(τ) + log(700))] (and thus, asymptotic sizes) were estimated to occur at SVL = 735 mm for slowest growing (τ = 0.05), SVL = 1160 mm for average growing (τ = 0.50), and SVL = 1515 mm for fastest growing (τ = 0.95) females. For males, equilibrium growth rates were estimated to occur at SVL = 835 mm for slowest growing (τ = 0.05), SVL = 1321 mm for average growing (τ = 0.50), and SVL = 1856 mm for fastest growing (τ = 0.95) snakes.

Most Brown Treesnakes on Guam do not show morphological evidence of maturation until 910–1025 mm SVL (females) and 940–1030 mm SVL (males) (Savidge et al. 2007). Assuming a male matures at 985 mm SVL, the 50th growth percentile (and 5th, 95th growth percentile) male snakes in our study population might become sexually mature at an age of 33 (∞, 15) months, where ∞ indicates slowest growing snakes never attain this size. The corresponding female age at maturation is 41 (∞, 18) months if maturing at 968 mm SVL. Age at maturity might be as soon as 10.4 (males) versus 13.7 (females) months should some fast‐growing (95th percentile) individuals mature at 805 versus 840 mm SVL (Savidge et al. 2007). These calculations assume a hatchling size of 350 mm SVL. However, hatchling Brown Treesnakes on Guam may be as small as 290 mm SVL (Lardner et al. 2009) or close to 430 mm SVL (Rodda, Fritts, et al. 1999), and in such cases age at maturation may be affected correspondingly.

Our growth increment form of von Bertalanffy models in their linear estimation form did not explain near as much variation in growth rates as Gompertz models, with *R* ^1^ coefficients of determination that were only 60% (lower quantiles) to 5% (higher quantiles) as large as the linearized version of comparable Gompertz growth models (Figure S4). Estimates of asymptotic size where growth rates equilibrate in von Bertalanffy models were unreasonably large for higher quantiles (Figure S4C,D). Estimated sizes with equilibrium growth rates were SVL = 2849 and 3868 mm for τ = 0.90 and 0.95, respectively, for females and SVL = 3525 and 4836 mm for τ = 0.90 and 0.95, respectively, for males. We do not consider the von Bertalanffy model an adequate approximation compared to the Gompertz model and do not consider it for further analyses.

When adding to the Gompertz model cumulative precipitation over 90 days, lagged between 0 and 51 weeks, the model with a lag of 1 week had most support in terms of median ∆BIC across quantiles, followed by a lag of 6 weeks (Figure S5). The model with a 1‐week lag suggested that snakes grew faster the more it had rained (Figure S5). When using a sinusoidal seasonality instead of precipitation, the Gompertz quantile regression models with strongest support (based on median ∆BIC across quantiles) had seasonality lagged with either 25 or 51 weeks (Figure S6A). These models indicated snakes grew fastest in September—October and, conversely, slowest in March—April (Figure S6C). As coefficients for 25‐ and 51‐week lags are perfectly negatively correlated, we only considered the 25‐week seasonal lag model.

**FIGURE 3:**
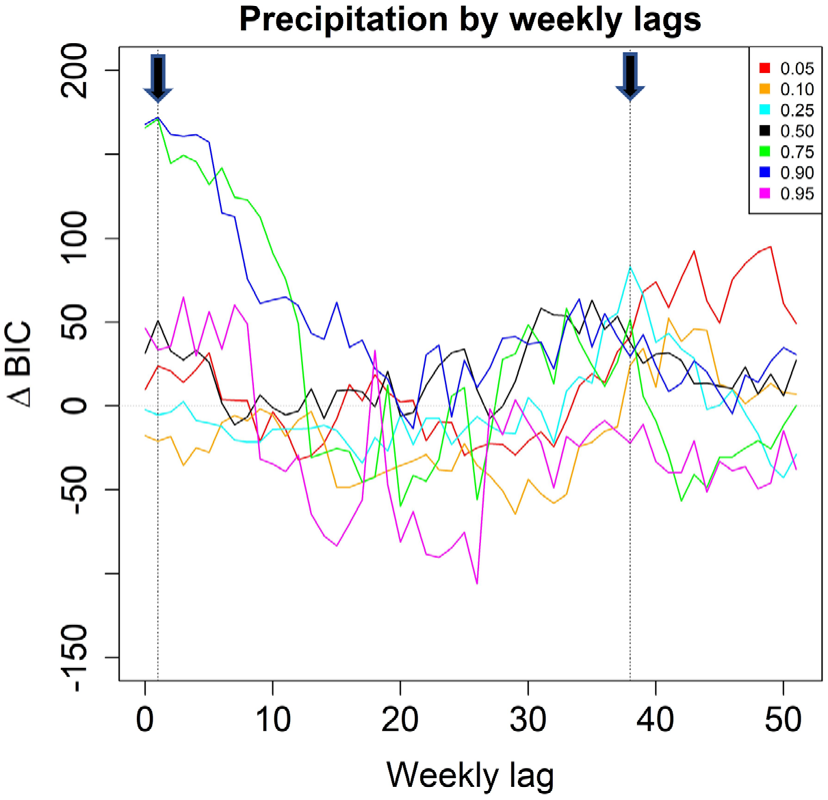
Differences in Bayesian Information Criterion (BIC) between the Brown Treesnake growth rate models in Guam that included only the logarithm of prior SVL (SVL_*i*, *j*_) (simple Gompertz growth model with separate intercepts for males and females, with least absolute shrinkage and selection operator (LASSO) shrinkage of intercepts for individual snakes) and models that also included seasonality with a 25‐week lag interacting with precipitation accumulating over 90 days at different weekly lags. Vertical lines denoted with black arrows indicate the weekly lagged values for which we screened partial estimates and 95% confidence intervals by quantile.

**FIGURE 4:**
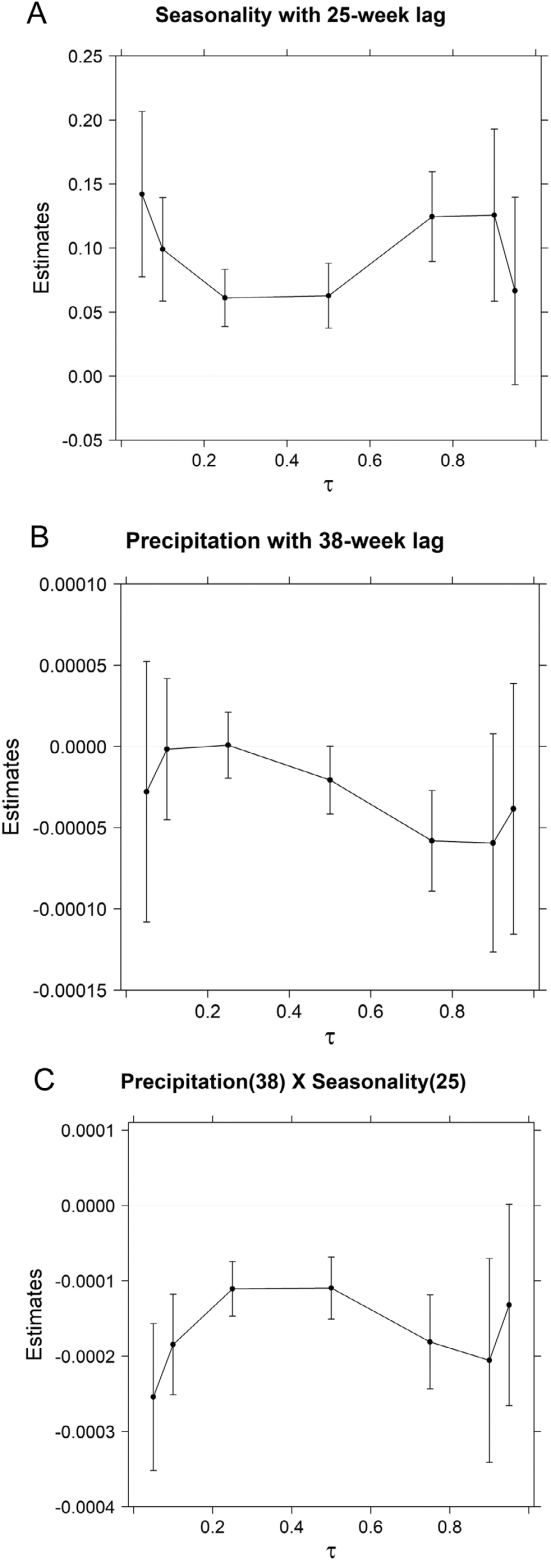
Parameter estimates and 95% confidence intervals for linear quantile regression (τ = {0.05, 0.10, 0.25, 0.50, 0.75, 0.90, 0.95}) models of male (*n* = 1517 for 142 individuals) and female (*n* = 1302 for 128 individuals) Brown Treesnake growth rates in Guam in a Gompertz model of logarithm of prior snout‐vent length (SVL_*i*, *j*_), with common slopes for males and females) that includes cyclical seasonality with a 25‐week lag (A), 90‐day cumulative precipitation with a 38‐week lag (B), and the interaction of these two terms (C).

**FIGURE 5:**
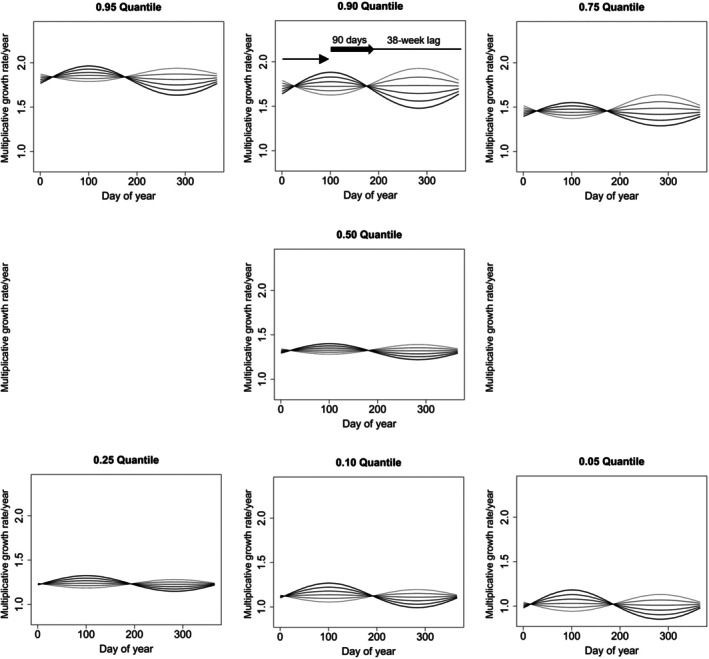
Model predicted annual growth rates in Brown Treesnakes on Guam as a function of the interactive effects of cumulative rainfall and seasonality when rainfall was measured over 90 days and lagged 38 weeks and when seasonality is lagged 25 weeks. This is a Gompertz model where both sexes change similarly with logarithm of prior snout‐vent length (SVL_*i*, *j*_), but sexes have differing intercepts scaled to equal log (700 mm). All models included the least absolute shrinkage and selection operator (LASSO) shrinkage of separate intercepts for individual snakes. Shown here are model predictions for females with prior SVL = 700 mm. For each of seven growth quantiles (indicated in each panel), model predictions were made for six different amounts of cumulative rainfall, with thicker lines indicating higher values: 200, 400, 600, 800, 1000, and 1200 mm. At other values of SVL, and/or for males, the pattern of changes by day of year (where zero equals 01 January) and precipitation will be identical by quantiles although the specific values would change. In the upper center panel, the thick black arrow indicates the 90‐day time frame over which rain accumulated and thin black line its 38‐week lag for the point in time in the following year when snakes would be most affected by that rainfall.

When including precipitation over 90 days at alternative lags and its interaction with the selected 25‐week seasonal lag, two alternative lags of 90RAIN were considered further (Figure 3). The lag of 38 weeks for 90‐day precipitation was most strongly supported, and there was little confounding correlation between the rain and season variables (*r* = 0.07). Estimated coefficients for seasonality, precipitation, and their interactions are in Figure 4. Partial estimates for females at a prior SVL = 700 mm for this model indicated higher growth rates when rainfall has been less seasonal than usual—that is, if the preceding wet season (almost 1 year earlier) had been unseasonably dry and if the preceding dry season (almost 1 year earlier) had been unseasonably wet (Figure 5). For example, a fast‐growing snake at the 0.90 quantile at SVL = 700 mm in early April would have an annual proportionate growth rate of 1.63 if precipitation was 200 mm, increasing to 1.68 if precipitation was instead 400 mm in the relevant 90‐day period of the previous dry season (Figure 5). The lag of 1 week for 90‐day precipitation was slightly less supported in terms of BIC, and there was a high (and potentially confounding) correlation (*r* = 0.81) between the rain and season variables. Estimated coefficients are in Figure S7. Partial estimates for females at a prior SVL = 700 mm for this model indicated a season‐by‐rainfall interaction pattern quite different from that in the model with 90RAIN lagged 38 weeks: higher growth rates when the current dry season (March–April) is unusually dry and when the current wet season (September–October) is unusually wet (Figure S8).

**FIGURE 6:**
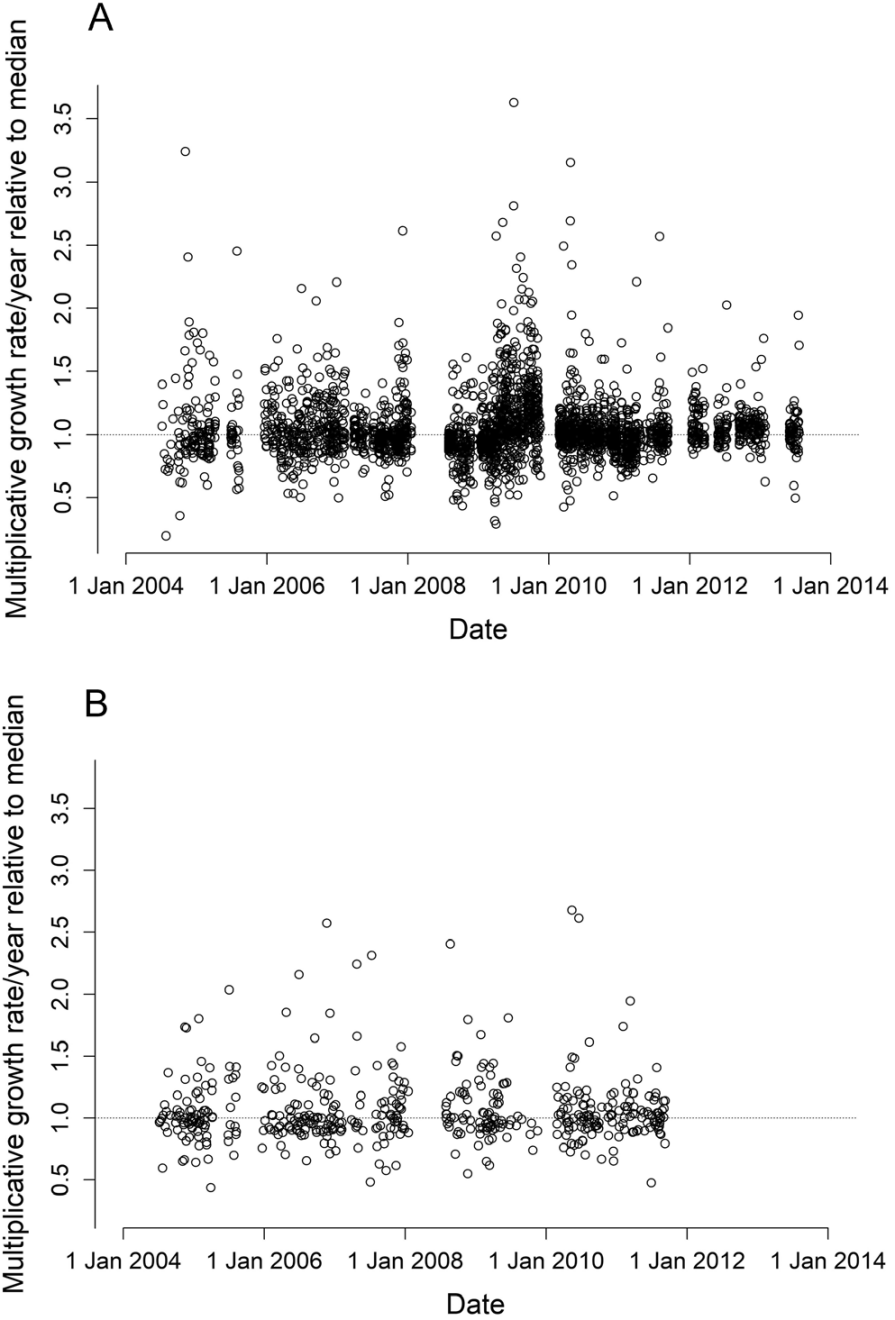
Multiplicative growth rates with respect to median (τ = 0.50) estimates from Gompertz model with separate intercepts for males and females and least absolute shrinkage and selection operator (LASSO) shrinkage for individual snake intercepts for Brown Treesnakes in Guam. Estimates were obtained by exponentiating residuals from the linear version of Gompertz model and graphed by date of observation. Panel (A) shows estimates from snakes of all sizes (*n* = 2819); panel (B) shows estimates obtained at less than 900 mm snout‐vent length (SVL) (*n* = 1091).

Neither of the two models with cyclical seasonality and its interaction with 90‐day precipitation (lagged either 38 or 1 weeks) explained substantially more variation in Brown Treesnake growth rates, as their inclusion only increased *R* ^1^ coefficients of determination by 0.001–0.027 over those for Gompertz models without these environmental covariates. This is consistent with our observations that both lower and higher growth rates for Brown Treesnakes are occurring at the same time in this restricted geographic location with no obvious trends across years (Figure 6).

## 4 Discussion

Methods for analyzing organismal growth have been a focus of research for more than a century (von Bertalanffy 1938, and references therein), and the conventional von Bertalanffy growth function is still widely used (e.g., Nelson 2014; Connette et al. 2015; Nafus et al. 2022). However, the von Bertalanffy growth function is only one of many nonlinear growth functions that asymptote to zero rate of change (Goshu and Koya 2013) and need not always serve as a reasonable approximation of growth. Our Gompertz models of Brown Treesnake growth on Guam were superior approximations to comparable von Bertalanffy models. By estimating quantiles of growth and incorporating individual snakes in our model, we were able to capture substantial variation in growth ignored in other growth models. Males often grew faster than females, and there was substantial within‐sex variation in growth rate that was greater for faster growing males than females. Those growth rate differences occurred at the same (5‐ha) site and at the same points in time. Snakes grew a little faster during the wet season and there was weak evidence for growth being affected by an interaction between seasonality and rainfall—at least when it comes to snakes that grew at an average or fast rate. Our estimate of female Brown Treesnake size when growth equilibrates for individuals with average growth rates (τ = 0.50) was SVL = 1160 mm, which is slightly larger than the comparable size of SVL = 1073 mm estimated for female Brown Treesnakes using a von Bertalanffy growth function (Nafus et al. 2022). We were able to capture greater variation among individuals of both sexes than Nafus et al. (2022) both because of our use of quantiles and by including a greater range of individual snake measurements in our models.

A limitation of our growth modeling of Brown Treesnakes was that some unknown amount of measurement error occurred, as suggested by the frequent occurrence of logarithms of growth rates less than zero, even though we restricted analyses to measures of SVL taken ≥ 20 days apart. Although these declining growth rates might appear to influence primarily estimates of lower quantiles (τ = 0.05 and 0.10) in our Gompertz models, there is reason to assume that an equal amount of measurement error is associated with the higher growth rate estimates. Therefore, asymptotic sizes of Brown Treesnakes for both the highest and the lowest quantiles warrant caution. It also means that our estimates of how long it may take slow‐ and fast‐growing snakes to mature are likely to be somewhat over‐ versus underestimated. We can get a crude estimate of potential measurement error by taking the absolute value of half the negative growth increments, which assumes both larger and smaller SVL measures may be in error and that the average between them is the best estimate of SVL. These ranged from a minimum = 0.5 mm to maximum = 62 mm with a median = 6.5 mm (*n* = 413). Converted to a percentage of the focal snake's size at the time, the median of these errors was 0.6% of the snake's snout‐vent length (min = 0.04%, max = 4.70%).

In our dataset, no female exceeded 1251 mm SVL whereas in a sample of 816 female Brown Treesnakes from across Guam (Table 1.4 in Siers 2015) the largest individual was 1475 mm SVL. Hence, our modeled asymptotic size of 1515 mm SVL in the fastest growing (τ = 0.95) females appears reasonable for Guam as a whole, but surprisingly large given the data we based our model on. Similarly, our modeled male asymptotic size of 1856 mm SVL in the fastest growing (τ = 0.95) males by far exceeded the size of our largest male (1477 mm SVL). However, the largest of the 992 male Brown Treesnakes sampled by Siers (2015) was 1950 mm SVL. We suspect that the aforementioned measurement errors may have contributed to the fact that our modeled max‐size asymptotes exceeded the size of our largest female and male snakes—even if snakes exist on Guam that better fit the model predicted max‐sizes. There have been no large‐scale capture‐mark‐recapture of free‐roaming Brown Treesnakes on Guam that allow an in‐depth analysis of growth rate variation across sites and over time, but data on ca 1800 snakes collected from 18 sites of six habitat classes across the island suggest a profound spatio‐temporal variation in body conditions and size distributions (Siers et al. 2017). In forested habitats on Guam, Brown Treesnakes usually occur in densities high enough to suppress the rodent populations (Rodda et al. 1999; Nafus et al. 2024). If a snake management program (i.e., culling) reduces the local snake density significantly, the rat population will increase (Nafus et al. 2024) and, as a side effect, any adult, rat‐eating snakes will have a higher likelihood of foraging success, a higher body condition, a faster growth rate, and a larger asymptotic size (unless removed by the management program before the potential asymptotic size is realized).

Even though measurement errors may have somewhat inflated the range of growth rates and asymptotic sizes, screening of data on individual snakes' size over time suggests some were approaching asymptotic sizes so small that they might never become sexually mature. However, we note that growth can (and sometimes does, from inspection of individual snakes' growth trajectories) increase after a period of arrested growth. For none of the snakes in which growth seemed to come to a halt at a very small size do we have data extending for a long time thereafter. It is possible that they resumed growing when conditions improved (and that they eventually reached a mature size), or that they died or were removed by us for experimental reasons, and therefore had no opportunity to resume growing.

Snakes tended to grow fastest during the wet season, and in an alternative model there was a positive correlation between cumulative rain over 90 days lagged for 1 week. On their own, these models are flip‐sides of the same coin. If snake activity (and foraging) correlates positively to growth, these findings agree with movements (road crossing rate) of radio‐tracked Brown Treesnakes being positively correlated to humidity and tending to correlate positively to rainfall (Siers et al. 2016).

While our results suggest that less extreme seasonal rainfall (drier wet and/or wetter dry seasons) may positively affect snake growth via direct or indirect pathways (cf. Sperry and Weatherhead 2008), we have not identified the mechanism(s) responsible. The fact that our most supported interaction model (which also did not suffer from collinearity between the SEASON and 90RAIN covariates) identified an effect of rainfall that was delayed with 38 weeks (after the 3 months during which cumulative rainfall was measured; see joint effect shown in Figure 5) suggests that a demographic response in prey populations may be involved (*sensu* Rose et al. 2018). In our study population, as well as in most non‐urban areas of Guam, the primary prey available to snakes were the geckos *Hemidactylus frenatus* and *Lepidodactylus lugubris* , the skinks *Carlia ailanpalai* and *Emoia caeruleocauda* and the rat *Rattus* cf. *diardii* (Wiewel et al. 2009; Campbell et al. 2012; Lardner et al. 2015; Siers 2015). Brown treesnakes on Guam undergo an ontogenetic transition in what they eat (generally speaking, this transition is from ectotherms to endotherms; Savidge 1988; Siers 2015), and we have no knowledge of how seasonal extremes affect the relevant prey populations in our system but acknowledge that such effects have been observed elsewhere (Madsen et al. 2006; Ujvari et al. 2011). Teasing out which prey taxa may have affected our model outcome would therefore be very difficult.

Had there been more merit in the interaction model that indicated a mere 1‐week lag in the 90RAIN effect (the collinearity between the covariates of which raised concern, and it was less supported by BIC‐statistics), the short lag would have seemed to indicate a behavioral response (of snakes, their prey, or both) rather than a demographic prey response.

Although the magnitude of partial estimates in Figure 5 suggests rather strong effects of precipitation on growth rates, the amount of additional growth rate variation explained by these environmental covariates was minimal. Also, one might consider in Figure 5 that a snake might jump between different precipitation curves over the course of a year, which would tend to reduce the overall seasonal fluctuation in its growth (cf. Figure S5C). This suggests that although statistically verified, the magnitude of seasonal/rainfall effects is small compared to the unexplained between‐snake and within‐sex variation.

We observed noticeable differences in growth rates among snakes at the same study site during the same points in time (Figure 6). Especially for snakes < 900 mm SVL, which feed primarily on lizards (Savidge 1988; Siers 2015), this simultaneous among‐snake growth variation by far exceeded any temporal growth fluctuations (Figure 6B). Trade‐offs between growth rate and other fitness‐related traits mean that slow growth is not necessarily indicative of a poor‐quality phenotype (Arendt 1997; Mangel and Stamps 2001; Metcalfe and Monaghan 2001). Also, we have no data linking growth rates to variation in age or size at maturity, both of which may have fitness consequences (Seigel and Ford 1987; Shine 2003). Even if fast‐growing individuals mature at a younger age, the size at which they mature is difficult to predict (Byars et al. 2010).

Sex‐differentiated allocation of resources often becomes evident at maturation, prior to which juvenile males and females have similar growth trajectories (Diller and Wallace 2002; Taylor and Denardo 2005). But among our snakes, the average female grew slower than the average male from the time they hatched (Figure 1). In visual encounter surveys using capture‐mark‐recapture, detection probability for juvenile females is lower than for juvenile males (Christy et al. 2010). Perhaps males are more active and less risk averse than females (Wolf and Weissing 2012), which could enhance their foraging success and increase growth over that of females (cf. Taylor and Denardo 2005) while making them more exposed to predators (and biologists). We have yet to analyze the trade‐off between growth and reproductive investment in adult Brown Treesnake females, but we know that males can attain much larger sizes than females (Savidge 1991; Rodda et al. 1999; this study).

Adaptive hypotheses for growth rate variation notwithstanding (cf. Bronikowski 2000), it is worth noting that the Brown Treesnake population on Guam was founded less than 75 years ago (Rodda and Savidge 2007), presumably by less than 10 snakes and possibly by a single gravid female (Richmond et al. 2015). It is conceivable that differential inbreeding coefficients among snakes play a role in the large within‐sex growth variation we observed (cf. Madsen et al. 1996, 1999; Crnokrak and Roff 1999; Hedrick and Kalinowski 2000). This also raises the question: if we suspect a particular trait expression to be adaptive, in which environment—native or novel—would it maximize fitness?

### 4.1 Management Implications

Our understanding of growth is limited for most species. When estimated, growth is often described by monotonically decreasing models such as the von Bertalanffy model, which in traditional applications fails to account for individual variability; this is problematic because such variability can greatly affect model results (Wang and Ellis 1998). Our quantile regression implementation of Gompertz growth models accounts for much greater individual variability in growth. Just as data on growth and reproduction can inform management efforts for species of conservation concern, they can inform control efforts for invasive species such as Brown Treesnakes. Current Brown Treesnake control methods using mice as lures or baits can suppress the segment of the population consisting of snakes of medium and large sizes but are largely ineffective at targeting small juveniles (Rodda et al. 2007; Tyrrell et al. 2009; Lardner et al. 2013). Our data on growth rate variation can be used to improve modeling efforts for predicting efficacy of snake control programs (e.g., Nafus et al. 2020; Siers et al. 2020). An example would be to estimate the total duration of a control program required to ensure that eggs or newborn snakes have time to grow to a size at which they all are susceptible to control; another is modeling maximal duration of the temporal window between control bouts without risking that snakes previously too small for capture have grown to reproductive size, by calculating joint probability distributions. Managers and decision makers will then need to balance the risk of failing to reach control goals against the cost of the associated management efforts. Snake control on a landscape scale is expensive but taking no action may eventually incur much higher costs (Shwiff et al. 2010). Our growth models have the potential to optimize the timing of control, thereby enhancing the cost‐effectiveness of population suppression or eradication efforts.

## Author Contributions

**Björn Lardner:** conceptualization (equal), data curation (equal), formal analysis (supporting), funding acquisition (supporting), methodology (equal), writing – original draft (lead), writing – review and editing (lead). **Brian S. Cade:** formal analysis (lead), methodology (supporting), writing – original draft (lead), writing – review and editing (lead). **Julie A. Savidge:** funding acquisition (supporting), methodology (equal), writing – original draft (supporting), writing – review and editing (supporting). **Gordon H. Rodda:** conceptualization (equal), funding acquisition (equal), methodology (equal), writing – original draft (supporting), writing – review and editing (supporting). **Robert N. Reed:** funding acquisition (equal), methodology (equal), writing – original draft (supporting), writing – review and editing (supporting). **Amy A. Yackel Adams:** conceptualization (equal), data curation (equal), formal analysis (supporting), funding acquisition (equal), project administration (lead), writing – original draft (lead), writing – review and editing (lead).

## Conflicts of Interest

The authors declare no conflicts of interest.

## Supporting information


Figure S1.

## Data Availability

The data and code used in this study necessary to reproduce our analysis are archived on the U.S. Geological Survey's ScienceBase (Lardner et al. 2025; https://doi.org/10.5066/P13NGCQS).

## References

[ece371695-bib-0001] Arendt, J. D. 1997. “Adaptive Intrinsic Growth Rates: An Integration Across Taxa.” Quarterly Review of Biology 72: 149–177.

[ece371695-bib-0002] Armstrong, D. P. , and R. J. Brooks . 2013. “Application of Hierarchical Biphasic Growth Models to Long‐Term Data for Snapping Turtles.” Ecological Modelling 250: 119–125.

[ece371695-bib-0003] Blouin‐Demers, G. 2003. “Precision and Accuracy of Body‐Size Measurements in a Constricting, Large‐Bodied Snake (*Elaphe obsoleta*).” Herpetological Review 34: 320–323.

[ece371695-bib-0004] Blouin‐Demers, G. , K. A. Prior , and P. J. Weatherhead . 2002. “Comparative Demography of Black Rat Snakes ( *Elaphe obsoleta* ) in Ontario and Maryland.” Journal of Zoology 256: 1–10.

[ece371695-bib-0005] Bronikowski, A. M. 2000. “Experimental Evidence for the Adaptive Evolution of Growth Rate in the Garter Snake *Thamnophis elegans* .” Evolution 54: 1760–1767. 11108602 10.1111/j.0014-3820.2000.tb00719.x

[ece371695-bib-0006] Byars, D. J. , N. B. Ford , A. M. Sparkman , and A. M. Bronikowski . 2010. “Influences of Diet and Family on Age of Maturation in Brown House Snakes, *Lamprophis fuliginosus* .” Herpetologica 66: 456–463.

[ece371695-bib-0007] Cade, B. S. , D. R. Edmunds , and D. S. Ouren . 2022. “Quantile Regression Estimates of Animal Population Trends.” Journal of Wildlife Management 86: e22228.

[ece371695-bib-0008] Cade, B. S. , and B. R. Noon . 2003. “A Gentle Introduction to Quantile Regression for Ecologists.” Frontiers in Ecology and the Environment 1: 412–420.

[ece371695-bib-0009] Cade, B. S. , J. W. Terrell , and B. C. Neely . 2011. “Estimating Geographic Variation in Allometric Growth and Body Condition of Blue Suckers With Quantile Regression.” Transactions of the American Fisheries Society 140: 1657–1669. 10.1080/00028487.2011.641885.

[ece371695-bib-0010] Cade, B. S. , J. W. Terrell , and M. T. Porath . 2008. “Estimating Fish Body Condition With Quantile Regression.” North American Journal of Fisheries Management 28: 349–359. 10.1577/M07-048.1.

[ece371695-bib-0011] Campbell, E. W. , A. A. Yackel Adams , S. J. Converse , T. H. Fritts , and G. H. Rodda . 2012. “Do Predators Control Prey Species Abundance? An Experimental Test With Brown Treesnakes on Guam.” Ecology 93: 1194–1203. 10.1890/11-1359.1. 22764505

[ece371695-bib-0012] Casey, J. 2021. “Climate Charts. Guam/Mariana Is., United States of America (Pacific Islands).” Accessed 23 February 2021. https://www.climate‐charts.com/Locations/u/U191212.html.

[ece371695-bib-0013] Christy, M. T. , A. A. Yackel Adams , G. H. Rodda , J. A. Savidge , and C. L. Tyrrell . 2010. “Modelling Detection Probabilities to Evaluate Management and Control Tools for an Invasive Species.” Journal of Applied Ecology 47: 106–113.

[ece371695-bib-0014] Connette, G. M. , J. A. Crawford , and W. E. Peterman . 2015. “Climate Change and Shrinking Salamanders: Alternative Mechanisms for Changes in Plethodeontid Salamander Body Size.” Global Change Biology 21: 2834–2843. 25641384 10.1111/gcb.12883

[ece371695-bib-0015] Crnokrak, P. , and D. A. Roff . 1999. “Inbreeding Depression in the Wild.” Heredity 83: 260–270. 10504423 10.1038/sj.hdy.6885530

[ece371695-bib-0016] Dennis, B. , J. M. Ponciano , M. L. Taper , and S. R. Lele . 2019. “Errors in Statistical Inference Under Model Misspecification: Evidence, Hypothesis Testing, and AIC.” Frontiers in Ecology and Evolution 7: 372–400. 10.3389/fevo.2019.00372. 34295904 PMC8293863

[ece371695-bib-0017] Diller, L. V. , and R. L. Wallace . 2002. “Growth, Reproduction, and Survival in a Population of *Crotalus viridis Oreganus* in North Central Idaho.” Herpetological Monographs 16: 26–45.

[ece371695-bib-0018] Dunham, A. E. 1978. “Food Availability as a Proximate Factor Influencing Individual Growth Rates in the Iguanid Lizard *Sceloporus merriami* .” Ecology 59: 770–778.

[ece371695-bib-0019] Eaton, M. J. , and W. A. Link . 2011. “Estimating Age From Recapture Data: Integrating Incremental Growth Measures With Ancillary Data to Infer Age‐at‐Length.” Ecological Applications 21: 2487–2497. 22073638 10.1890/10-0626.1

[ece371695-bib-0020] Fritts, T. H. , and G. H. Rodda . 1998. “The Role of Introduced Species in the Degradation of Island Ecosystems: A Case History of Guam.” Annual Review of Ecology and Systematics 29: 113–140.

[ece371695-bib-0021] Goshu, A. T. , and P. R. Koya . 2013. “Derivation of Inflection Points of Nonlinear Regression Curves—Implications to Statistics.” American Journal of Theoretical and Applied Statistics 2: 268–272.

[ece371695-bib-0022] Hart, D. R. , and A. S. Chute . 2009. “Estimating von Bertalanffy Growth Parameters From Growth Increment Data Using a Linear Mixed‐Effects Model, With an Application to the Sea Scallop *Placopecten magellanicus* .” ICES Journal of Marine Science 66: 2165–2175.

[ece371695-bib-0023] Hedrick, P. W. , and S. T. Kalinowski . 2000. “Inbreeding Depression in Conservation Biology.” Annual Review of Ecology and Systematics 31: 139–162.

[ece371695-bib-0024] Kirkpatrick, M. 1984. “Demographic Models Based on Size, Not Age, for Organisms With Indeterminate Growth.” Ecology 65: 1874–1884.

[ece371695-bib-0025] Klug, P. E. , A. A. Yackel Adams , and R. N. Reed . 2021. “Olfactory Lures in Predator Control Do Not Increase Predation Risk to Birds in Areas of Conservation Concern.” Wildlife Research 49: 183–192.

[ece371695-bib-0026] Koenker, R. 2005. Quantile Regression. Cambridge University Press.

[ece371695-bib-0027] Koenker, R. 2011. “Additive Models for Quantile Regression: Model Selection and Confidence Bandaids.” Brazilian Journal of Probability And Statistics 25: 239–262.

[ece371695-bib-0028] Koenker, R. 2014. “Package ‘quantreg’. Quantile Regression and Related Methods.” Accessed 29 October 2014. http://cran.r‐project.org/web/packages/quantreg/quantreg.pdf.

[ece371695-bib-0029] Koenker, R. , and J. A. F. Machado . 1999. “Goodness of Fit and Related Inference Processes for Quantile Regression.” Journal of the American Statistical Association 94: 1296–1310.

[ece371695-bib-0030] Lardner, B. , B. S. Cade , J. A. Savidge , G. H. Rodda , R. N. Reed , and A. A. Yackel Adams . 2025. “Growth Rate of Brown Treesnakes (*Boiga irregularis*) and R Code, Guam 2004–2013.” U.S. Geological Survey data Release. 10.1002/ece3.71695 PMC12259306 40661908

[ece371695-bib-0031] Lardner, B. , G. H. Rodda , A. A. Yackel Adams , J. A. Savidge , and R. N. Reed . 2015. “Detection Rates of Geckos in Visual Surveys: Turning Confounding Variables Into Useful Knowledge.” Journal of Herpetology 49: 522–532. 10.1670/14-048.

[ece371695-bib-0032] Lardner, B. , J. A. Savidge , G. H. Rodda , and R. N. Reed . 2009. “Prey Preferences and Prey Acceptance in Juvenile Brown Treesnakes (*Boiga irregularis*).” Herpetological Conservation and Biology 4: 313–323.

[ece371695-bib-0033] Lardner, B. , A. A. Yackel Adams , J. A. Savidge , G. H. Rodda , R. N. Reed , and C. S. Clark . 2013. “Effectiveness of Bait Tubes for Brown Treesnake Control on Guam.” Wildlife Society Bulletin 37: 664–673.

[ece371695-bib-0034] Luiselli, L. 2005. “Snakes Don't Shrink, but ‘Shrinkage’ Is an Almost Inevitable Outcome of Measurement Error by the Experimenters.” Oikos 110: 199–202.

[ece371695-bib-0035] Madsen, T. , and R. Shine . 2001. “Do Snakes Shrink?” Oikos 92: 187–188.

[ece371695-bib-0036] Madsen, T. , R. Shine , M. Olsson , and H. Wittzell . 1999. “Restoration of an Inbred Adder Population.” Nature 402: 34–35.

[ece371695-bib-0037] Madsen, T. , B. Stille , and R. Shine . 1996. “Inbreeding Depression in an Isolated Population of Adders *Vipera berus* .” Biological Conservation 75: 113–118.

[ece371695-bib-0038] Madsen, T. , B. Ujvari , R. Shine , and M. Olsson . 2006. “Rain, Rats and Pythons: Climate‐Driven Population Dynamics of Predators and Prey in Tropical Australia.” Austral Ecology 31: 30–37. 10.1111/j.1442-9993.2006.01540.x.

[ece371695-bib-0039] Mangel, M. , and J. Stamps . 2001. “Trade‐Offs Between Growth and Mortality and the Maintenance of Individual Variation in Growth.” Evolutionary Ecology Research 3: 583–593.

[ece371695-bib-0040] Maritz, B. , and G. J. Alexander . 2011. “Morphology, Sexual Dimorphism, and Growth in the Smallest Viperid, *Bitis schneideri* (Reptilia: Squamata: Viperidae).” Journal of Herpetology 45: 457–462.

[ece371695-bib-0041] Metcalfe, N. B. , and P. Monaghan . 2001. “Compensation for a Bad Start: Grow Now, Pay Later?” Trends in Ecology & Evolution 16: 254–260. 11301155 10.1016/s0169-5347(01)02124-3

[ece371695-bib-0042] Nafus, M. G. , A. Reyes , T. Fies , and S. M. Goetz . 2024. “Adaptive Resource Management: Achieving Functional Eradication of Invasive Snakes to Benefit Avian Conservation.” Journal of Applied Ecology 61: 733–745. 10.1111/1365-2664.14597.

[ece371695-bib-0043] Nafus, M. G. , S. R. Siers , B. A. Levine , Z. C. Quiogue , and A. A. Y. Adams . 2022. “Demographic Response of Brown Treesnakes to Extended Population Suppression.” Journal of Wildlife Management 86: e22136. 10.1002/jwmg.22136.

[ece371695-bib-0044] Nafus, M. G. , A. A. Yackel Adams , S. M. Boback , S. R. Siers , and R. N. Reed . 2020. “Behavior, Size, and Body Condition Predict Susceptibility to Management and Reflect Post‐Treatment Frequency Shifts in an Invasive Snake.” Global Ecology and Conservation 21: e00834. 10.1016/j.gecco.2019.e00834.

[ece371695-bib-0045] Nelson, G. A. 2014. “Package ‘fishmethods’. Fishery Science Methods and Models in R. Manual Version 1.6‐0.” Accessed 29 October 2014. http://cran.r‐project.org/web/packages/fishmethods/fishmethods.pdf.

[ece371695-bib-0046] Nolan, E. T. , and J. R. Britton . 2019. “Spatial Variability in the Somatic Growth of Pikeperch *Sander lucioperca*, an Invasive Piscivorous Fish.” Ecology of Freshwater Fish 28: 330–340. 10.1111/eff.12456.

[ece371695-bib-0047] Paragamian, V. L. , and R. C. P. Beamesderfer . 2003. “Growth Estimates From Tagged White Sturgeon Suggest That Ages From Fin Rays Underestimate True Age in the Kootenai River, USA and Canada.” Transactions of the American Fisheries Society 132: 895–903.

[ece371695-bib-0048] Plummer, M. V. 1985. “Growth and Maturity in Green Snakes (*Opheodrys aestivus*).” Herpetologica 41: 28–33.

[ece371695-bib-0049] R Core Team . 2020. R: A Language and Environment for Statistical Computing. R Foundation for Statistical Computing.

[ece371695-bib-0050] Richmond, J. Q. , D. A. Wood , J. W. Stanford , and R. N. Fischer . 2015. “Testing for Multiple Invasion Routes and Source Populations for the Invasive Brown Treesnake (*Boiga irregularis*) on Guam: Implications for Pest Management.” Biological Invasions 17: 337–349.

[ece371695-bib-0051] Rodda, G. H. , T. H. Fritts , M. J. McCoid , and E. W. Campbell III . 1999. “An Overview of the Biology of the Brown Treesnake, *Boiga irregularis* , a Costly Introduced Pest on Pacific Islands.” In Problem Snake Management: The Habu and the Brown Treesnake, edited by G. H. Rodda , Y. Sawai , D. Chiszar , and H. Tanaka , 44–80. Cornell University Press.

[ece371695-bib-0052] Rodda, G. H. , M. J. McCoid , T. H. Fritts , and E. W. Campbell III . 1999. “Population Trends and Limiting Factors in Boiga Irregularis.” In Problem Snake Management: The Habu and the Brown Treesnake, edited by G. H. Rodda , Y. Sawai , D. Chiszar , and H. Tanaka , 236–253. Cornell University Press.

[ece371695-bib-0053] Rodda, G. H. , and J. A. Savidge . 2007. “Biology and Impact of Pacific Island Invasive Species.” Pacific Science 61: 307–324.

[ece371695-bib-0054] Rodda, G. H. , J. A. Savidge , C. L. Tyrrell , M. T. Christy , and A. R. Ellingson . 2007. “Size Bias in Visual Searches and Trapping of Brown Treesnakes on Guam.” Journal of Wildlife Management 71: 656–661.

[ece371695-bib-0055] Rogers, H. , E. R. Buhle , J. Hille Ris Lambers , E. C. Fricke , R. H. Miller , and J. J. Tewksbury . 2017. “Effects of an Invasive Predator Cascade to Plants via Mutualism Disruption.” Nature Communications 8: 14557. 10.1038/ncomms14557 PMC5344968 28270682

[ece371695-bib-0056] Rogers, H. , J. Hille Ris Lambers , R. Miller , and J. J. Tewksbury . 2012. “‘Natural Experiment’ Demonstrates Top‐Down Control of Spiders by Birds on a Landscape Level.” PLoS One 7: e43446. 22970126 10.1371/journal.pone.0043446 PMC3436874

[ece371695-bib-0057] Rose, J. P. , B. J. Halstead , G. D. Wylie , and M. L. Casazza . 2018. “Spatial and Temporal Variability in Growth of Giant Gartersnakes: Plasticity, Precipitation, and Prey.” Journal of Herpetology 52: 40–49.

[ece371695-bib-0058] Rose, J. P. , R. Kim , R. , et al. 2022. “Integrating Growth and Survival Models for Flexible Estimation of Size‐Dependent Survival in a Cryptic, Endangered Snake.” Ecology and Evolution 12: e8799. 10.1002/ece3.8799. 35414900 PMC8987119

[ece371695-bib-0059] Rose, J. P. , and B. D. Todd . 2020. “Targeting Eradication of Introduced Watersnakes Using Integral Projection Models.” Animal Conservation 23: 713–724.

[ece371695-bib-0060] Sakai, A. K. , F. W. Allendorf , J. S. Holt , et al. 2001. “The Population Biology of Invasive Species.” Annual Review of Ecology and Systematics 32: 305–332.

[ece371695-bib-0061] Savidge, J. A. 1987. “Extinction of an Island Forest Avifauna by an Introduced Snake.” Ecology 68: 660–668.

[ece371695-bib-0062] Savidge, J. A. 1988. “Food Habits of *Boiga irregularis*, an Introduced Predator on Guam.” Journal of Herpetology 22: 275–282.

[ece371695-bib-0063] Savidge, J. A. 1991. “Population Characteristics of the Introduced Brown Tree Snake (*Boiga irregularis*) on Guam.” Biotropica 23: 294–300.

[ece371695-bib-0064] Savidge, J. A. , F. J. Qualls , and G. H. Rodda . 2007. “Reproductive Biology of the Brown Tree Snake, *Boiga irregularis* (Reptilia: Colubridae), During Colonization of Guam and Comparison With That in Their Native Range.” Pacific Science 61: 187–195.

[ece371695-bib-0065] Schoener, T. W. , and A. Schoener . 1978. “Estimating and Interpreting Body‐Size Growth in Some *Anolis* Lizards.” Copeia 1: 390–405.

[ece371695-bib-0066] Seigel, R. A. , and N. B. Ford . 1987. “Reproductive Ecology.” In Snakes: Ecology and Evolutionary Biology, edited by R. A. Seigel , J. T. Collins , and S. S. Novak , 210–252. Macmillan.

[ece371695-bib-0067] Shine, R. 2003. “Reproductive Strategies in Snakes.” Proceedings of the Royal Society of London B 270: 995–1004. 10.1098/rspb.2002.2307 PMC1691341 12803888

[ece371695-bib-0068] Shine, R. , and E. L. Charnov . 1992. “Patterns of Survival, Growth, and Maturation in Snakes and Lizards.” American Naturalist 139: 1257–1269.

[ece371695-bib-0069] Shwiff, S. A. , K. Gebhardt , K. N. Kirkpatrick , and S. S. Shwiff . 2010. “Potential Economic Damage From Introduction of Brown Tree Snakes, *Boiga irregularis* (Reptilia: Colubridae), to the Islands of Hawai‘i.” Pacific Science 64: 1–10.

[ece371695-bib-0070] Siers, S. 2015. “Microgeographic and Ontogenetic Variability in the Ecology of Invasive Brown Treesnakes on Guam, and Effects of Roads on Their Landscape‐Scale Movements.” PhD Dissertation, Colorado State University, USA.

[ece371695-bib-0071] Siers, S. , R. N. Reed , and J. A. Savidge . 2016. “To Cross or Not to Cross: Modeling Wildlife Road Crossings as a Binary Response Variable With Contextual Predictors.” Ecosphere 7, no. 5: e01292. 10.1002/ecs2.1292.

[ece371695-bib-0072] Siers, S. , J. A. Savidge , and R. N. Reed . 2017. “Quantile Regression of Microgeographic Variation in Population Characteristics of an Invasive Vertebrate Predator.” PLoS One 12, no. 6: e0177671. 10.1371/journal.pone.0177671. 28570632 PMC5453442

[ece371695-bib-0073] Siers, S. R. , J. D. Eisemann , W. C. Pitt , et al. 2020. “Automated Aerial Baiting for Invasive Brown Treesnake Control: System Overview and Program Status.” Proceedings of the Vertebrate Pest Conference 29. https://escholarship.org/uc/item/0j58m801.

[ece371695-bib-0074] Sigourney, D. B. , S. B. Munch , and B. H. Letcher . 2012. “Combining a Bayesian Nonparametric Method With a Hierarchical Framework to Estimate Individual and Temporal Variation in Growth.” Ecological Modelling 247: 125–134.

[ece371695-bib-0075] Sohn, J. J. , and D. Crews . 1977. “Size‐Mediated Onset of Genetically Determined Maturation in the Platyfish, *Xiphophorus maculatus* .” Proceedings of the National Academy of Sciences of the United States of America 74: 4547–4548. 16592454 10.1073/pnas.74.10.4547 PMC431982

[ece371695-bib-0076] Sperry, J. H. , and P. J. Weatherhead . 2008. “Prey‐Mediated Effects of Drought on Condition and Survival of a Terrestrial Snake.” Ecology 89: 2770–2776. 18959314 10.1890/07-2017.1

[ece371695-bib-0077] Stanford, K. M. , and R. B. King . 2004. “Growth, Survival, and Reproduction in a Northern Illinois Population of the Plains Gartersnake, *Thamnophis radix* .” Copeia 2004: 465–478.

[ece371695-bib-0078] Taylor, E. N. , and D. F. Denardo . 2005. “Sexual Size Dimorphism and Growth Plasticity in Snakes: An Experiment on the Western Diamond‐Backed Rattlesnake (*Crotalus atrox*).” Journal of Experimental Biology 343: 598–607. 10.1002/jez.a.189 15945080

[ece371695-bib-0079] Tibshirani, R. 1996. “Regression Shrinkage and Selection via the Lasso.” Journal of the Royal Statistical Society, Series B: Statistical Methodology 58: 267–288.

[ece371695-bib-0080] Troynikov, V. S. , R. W. Day , and A. M. Leorke . 1998. “Estimation of Seasonal Growth Parameters Using a Stochastic Gompertz Model for Tagging Data.” Journal of Shellfish Research 17: 833–838.

[ece371695-bib-0081] Tyrrell, C. L. , M. T. Christy , G. H. Rodda , et al. 2009. “Evaluation of Trap Capture in a Geographically Closed Population of Brown Treesnakes on Guam.” Journal of Applied Ecology 46: 128–135.

[ece371695-bib-0082] Ujvari, B. , R. Shine , and T. Madsen . 2011. “How Well Do Predators Adjust to Climate‐Mediated Shifts in Prey Distribution? A Study on Australian Water Pythons.” Ecology 92: 777–783. 21608485 10.1890/10-1471.1

[ece371695-bib-0083] van Devender, R. W. 1978. “Growth Ecology of a Tropical Lizard, *Basiliscus basiliscus* .” Ecology 59: 1031–1038.

[ece371695-bib-0084] von Bertalanffy, L. 1938. “A Quantitative Theory of Organic Growth (Inquiries on Growth Laws. II).” Human Biology 10: 181–213.

[ece371695-bib-0085] Wang, Y.‐G. , and N. Ellis . 1998. “Effect of Individual Variability on Estimation of Population Parameters From Length‐Frequency Data.” Canadian Journal of Fisheries and Aquatic Sciences 55: 2393–2401.

[ece371695-bib-0086] Wiewel, A. S. , A. A. Yackel Adams , and G. H. Rodda . 2009. “Evaluating Abundance Estimate Precision and the Assumptions of a Count‐Based Index for Small Mammals.” Journal of Wildlife Management 73: 761–771.

[ece371695-bib-0087] Wolf, M. , and F. J. Weissing . 2012. “Animal Personalities: Consequences for Ecology and Evolution.” Trends in Ecology & Evolution 27: 452–461. 22727728 10.1016/j.tree.2012.05.001

