# Supplementary material for: Growth Rate Variation in Brown Treesnakes (Boiga irregularis): An Invasive Species of Conservation Concern
Source: Ecol Evol. 2025 Jul 14;15(7):e71695. doi: 10.1002/ece3.71695 (PMC12259306; doi:10.1002/ece3.71695)
Supplement: Supplementary file 1 — Figure S1. [file ECE3-15-e71695-s001.pdf]

# SUPPLEMENT

We here provide further information and figures associated with the paper "Growth rate variation in Brown Treesnakes (*Boiga irregularis*)".

Björn Lardner[1,2,3,a], Brian S. Cade[4,a], Julie A. Savidge[1], Gordon H. Rodda[4,5], Robert N. Reed[4,6], Amy A. Yackel Adams[4]

[1]*Department of Fish, Wildlife, and Conservation Biology, Colorado State University, Fort Collins, Colorado 80523, USA*

[2]*Corresponding author. E-mail: scinemato@gmail.com*

[3]Present address: *Bokekullsvägen 6C, 27730 Kivik, Sweden*

[4]*U.S. Geological Survey, Fort Collins Science Center, Fort Collins, Colorado 80526, USA*

[5]Present address: *404 Adobe Drive, Hesperus, Colorado 81326, USA*

[6]Present address: *U.S. Geological Survey, Pacific Island Ecosystems Research Center, Hawaii National Park, Hawaii 96718, USA*

[a]both authors contributed equally

**On missing precipitation data for one date**

Typhoon Tingting affected Guam on 27 – 28 June 2004 and led to a missing datum in the National Oceanic and Atmospheric Administration's (NOAA's) rainfall data for Guam International Airport. Wikipedia states (https://en.wikipedia.org/wiki/Typhoon_Tingting; accessed on 12 June 2022) that, "Although Tingting passed about 400 km (250 mi) to the northeast of Guam, torrential rains from a monsoonal feeder band produced record-breaking rainfall. In the span of 24 hours, 554.99 mm (21.85 in) of rain fell on Guam shattering both the record for highest single day rainfall and the monthly rainfall for June at 80.26 mm (3.160 in) and 371.09 mm (14.610 in) respectively. Most of the northern areas of the island received more than 510 mm (20 in) of rain but the southern areas of Guam reported much lower totals. At Anderson Air Force Base, a total of 287.78 mm (11.33 in) of rain was recorded over a 30-hour span." Further support for this large amount of rain comes from Gary Padgett's monthly global tropical cyclone summary web page for June 2004 (http://www.typhoon2000.ph/garyp_mgtcs/jun04sum.txt; accessed on 04 January 2022). It states that

according to meteorologist Mark Landers, who is based on Guam, “some stations in Guam received more than 600 mm in a 24-hour period”.

Most of the rain associated with Typhoon Tingting apparently fell during 27 June, but heavy rain continued on 28 June; NOAA data for Guam International Airport list 139 mm on 28 June. Because we cannot tell if the precipitation amounts mentioned in Wikipedia and elsewhere are for just one single date (i.e., 00:00 – 23:59 on 27 June) or for some arbitrary 24-hour period spanning both dates, we set the precipitation value for 27 June to (555 – 139) = 416 mm.

## Supplemental Figures

(A)

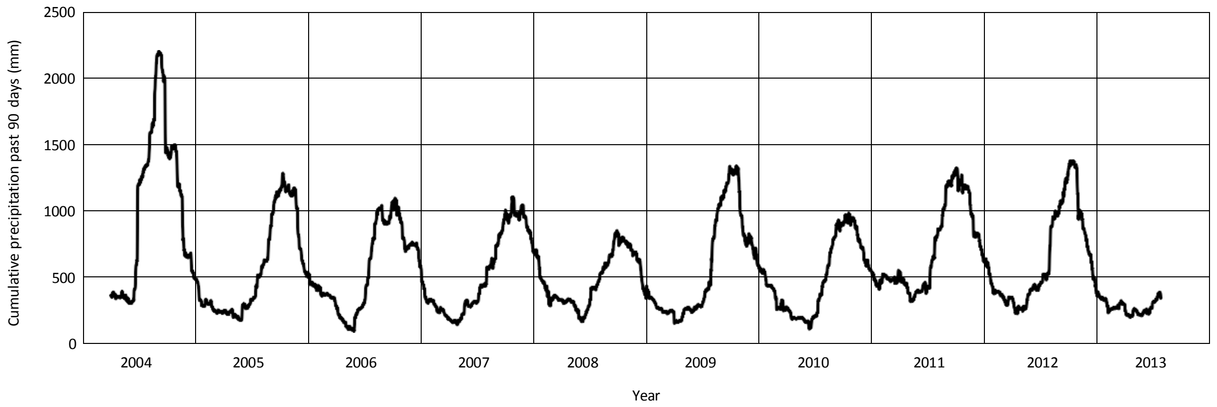


(B)

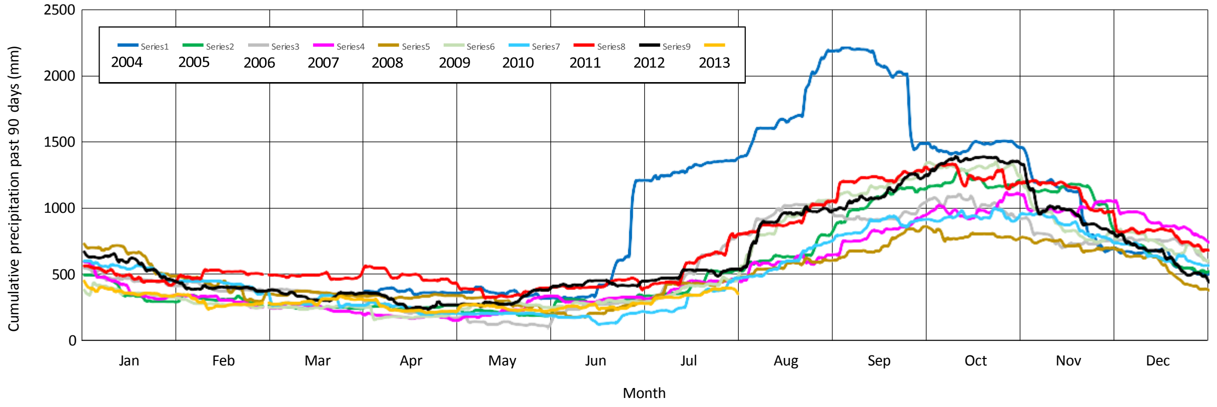


**Fig. S1.** Between-year and within-year variation in rainfall on Guam, measured as the cumulative amount (mm) of precipitation during the 90 days leading up to the focal date (in the manuscript, this variable is called 90RAIN). Shown here are data without any time lag, from 01 April 2004 to 31 July 2013. With the exception of 27 June 2004 (see previous page), raw data came from the National Oceanic and Atmospheric Administration's "NOWdata – NOAA Online Weather Data" (www.weather.gov/wrh/Climate?wfo=gum; accessed on 04 January 2022).

Females Males

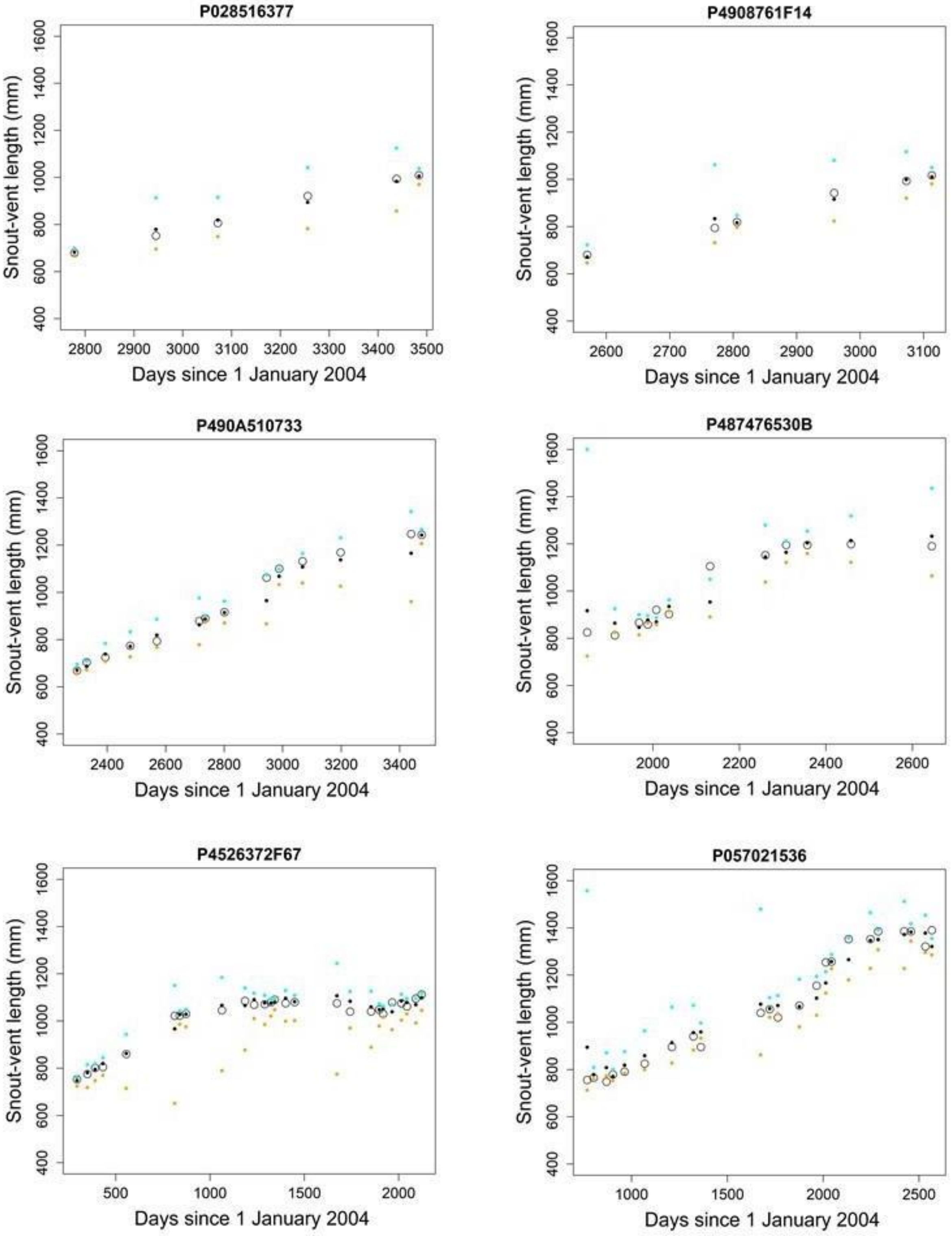

**FIG. S2.** Observed snout-vent length (SVL) by days since 1 January 2004 for 3 female and 3 male Brown Treesnakes (large open circles) measured in a 5-ha plot located in NW Guam. Individuals were selected to include those with sparse, moderate, and dense sampling of SVL over time consistent with range of variation among all individuals. Estimates for 0.05 (solid orange dots), 0.50 (solid black dots), and 0.95 (solid cyan dots) quantiles are shown for each individual from the Gompertz growth model that included least absolute shrinkage and selection operator (LASSO) shrinkage effect for individual snake intercepts. Snake P028516377 has 6 of 6 (100%), P4908761F14 has 6 of 6 (100%), P487476530B has 8 of 12 (75%), P490A510733 has 14 of 14 (100%), P4526372F67 has 25 of 26 (96%), and P057021536 has 18 of 23 (78%) of observed SVL within intervals formed by 0.05 and 0.95 quantile estimates.

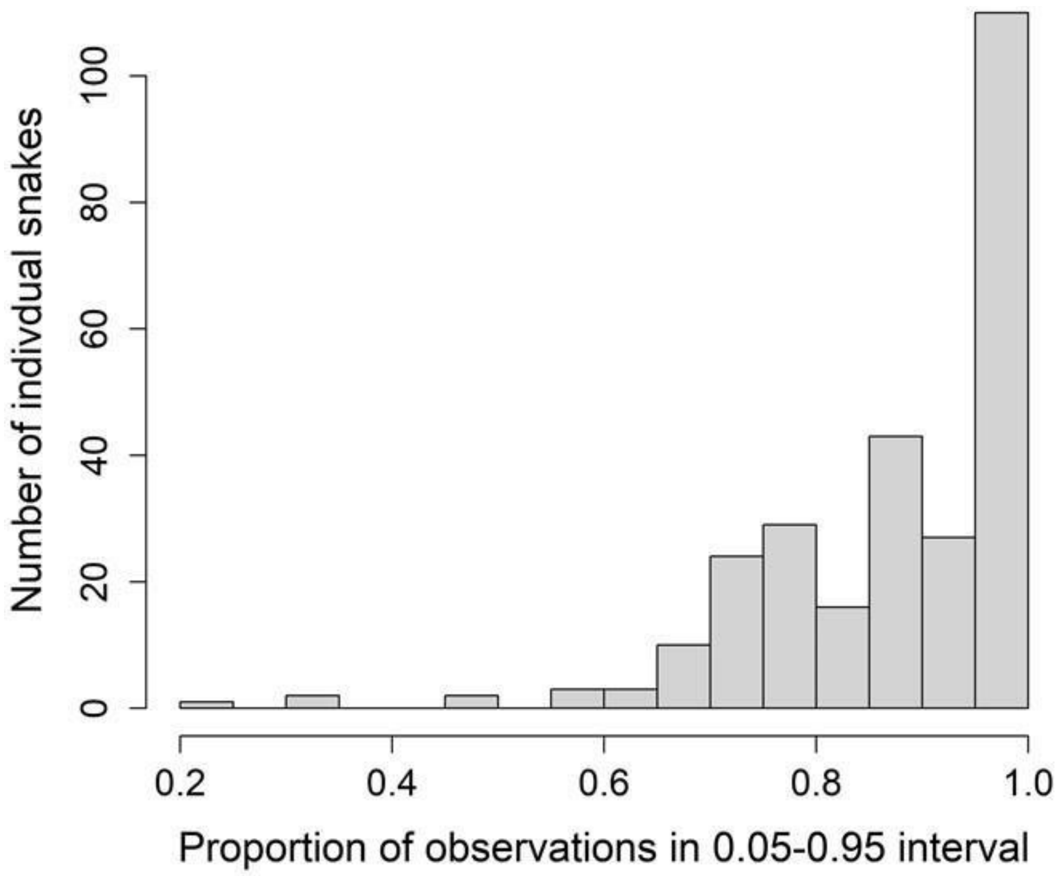


**FIG. S3.** Distribution of proportion of Brown Treesnake snout-vent length observations contained within intervals estimated by 0.05 and 0.95 quantiles for 270 individual snakes measured in a 5-ha plot located in NW Guam from 10 May 2004 to 18 July 2013. Quantile estimates were from Gompertz model with separate intercepts for males and females and least absolute shrinkage and selection operator (LASSO) shrinkage on individual snake intercepts.

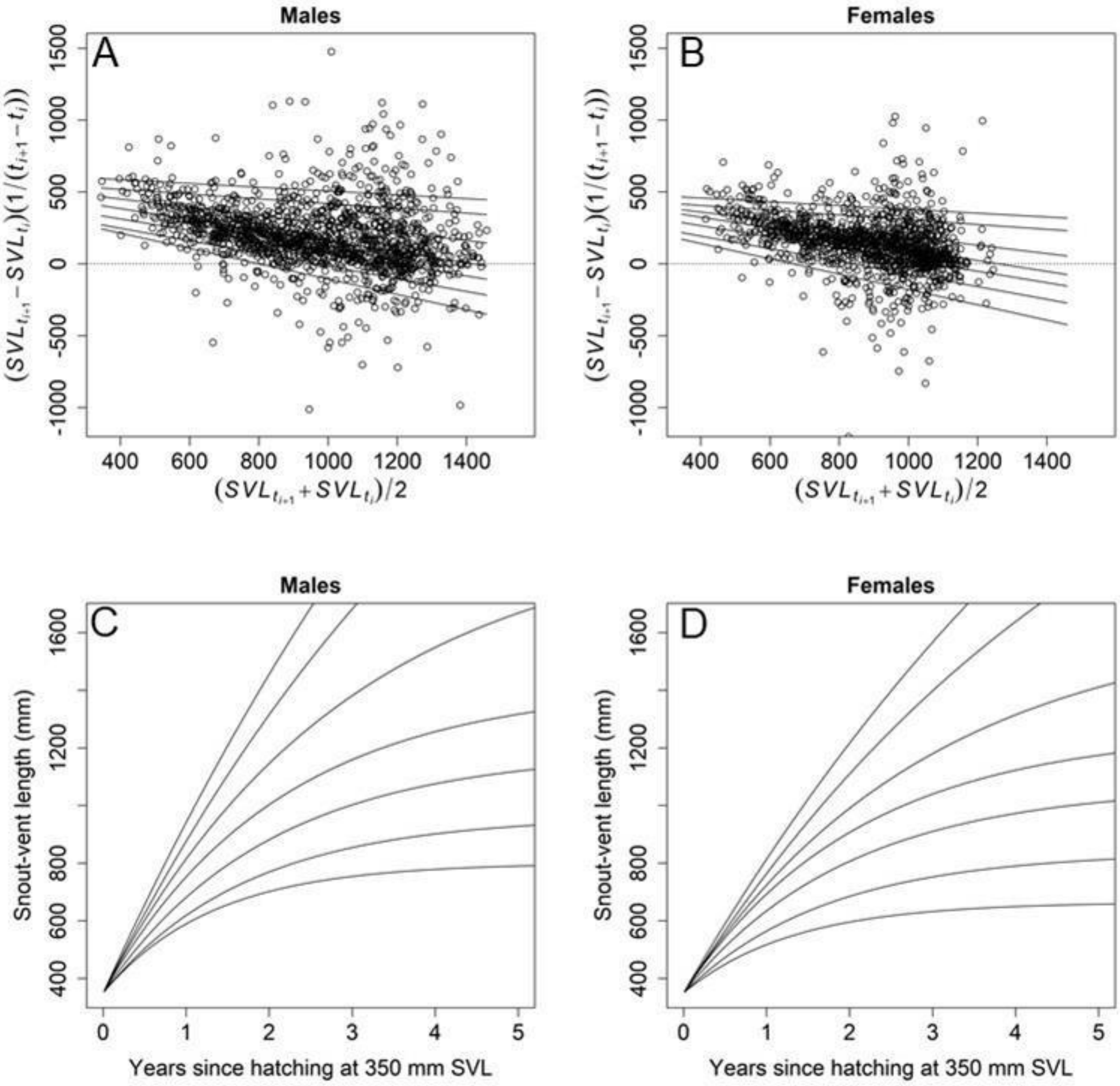


**FIG. S4.** Quantile regression ($\tau$ = {0.05, 0.10, 0.25, 0.50, 0.75, 0.90, 0.95}; lower to higher lines) estimates of juvenile male (*n* = 1,517 for 142 individuals) and female (*n* = 1,302 for 128 individuals) Brown Treesnake growth rates (mm/year) as an additive function of mid-point of snout-vent length (SVL) in a von Bertalanffy growth model using data collected in NW Guam from 10 May 2004 to 18 July 2013 Estimates were obtained from linear quantile regression models of annual growth rates in a model with common slopes but different intercepts for males (panel A) and females (panel B) with least absolute shrinkage and selection operator (LASSO) shrinkage on intercepts for individual snakes. Panels C and D are the corresponding multiplicative von Bertalanffy growth estimates made by starting growth at 350 mm SVL. The von Bertalanffy growth coefficients $K(\tau) = -(\log(\hat{\beta}_1(\tau) + 1))$ and asymptotes $SVL_\infty(\tau) = -\hat{\beta}_0(\tau)/\hat{\beta}_1(\tau)$, where $\hat{\beta}_0(\tau)$ and $\hat{\beta}_1(\tau)$ are estimates from linear quantile regression models in panels A and B. Growth coefficients *K*(0.95) = 0.142, *K*(0.90) = 0.181, *K*(0.75) = 0.341, *K*(0.50) = 0.474, *K*(0.25) = 0.505, *K*(0.10) = 0.579, and *K*(0.05) = 0.762 for both sexes. Asymptotes $SVL_\infty(0.95)$ = 4836 mm, $SVL_\infty(0.90)$ =

3525 mm , $SVL_\infty(0.75)$ = 1961 mm, $SVL_\infty(0.50)$ = 1417 mm , $SVL_\infty(0.25)$ = 1187 mm, $SVL_\infty(0.10)$ = 962 mm, and $SVL_\infty(0.05)$ = 800 mm for males and $SVL_\infty(0.95)$ = 3868 mm, $SVL_\infty(0.90)$ = 2849 mm, $SVL_\infty(0.75)$ = 1647 mm, $SVL_\infty(0.50)$ = 1260 mm, $SVL_\infty(0.25)$ = 1068 mm, $SVL_\infty(0.10)$ = 838 mm, and $SVL_\infty(0.05)$ = 664 mm for females. $R^1(\tau)$ coefficients of determination indicating the proportionate reduction in variation associated with adding the term mid-point of SVL to the linear quantile regression models with individual and sex effects were $R^1(0.95) = 0.011$, $R^1(0.90) = 0.022$, $R^1(0.75) = 0.052$, $R^1(0.50) = 0.091$, $R^1(0.25) = 0.100$, $R^1(0.10) = 0.081$, and $R^1(0.05) = 0.078$. These coefficients of determination indicated the linear approximation of the von Bertalanffy growth model only explained 60% (lower quantiles) to 5% (higher quantiles) as much variation as the linearized version of the comparable Gompertz growth models.

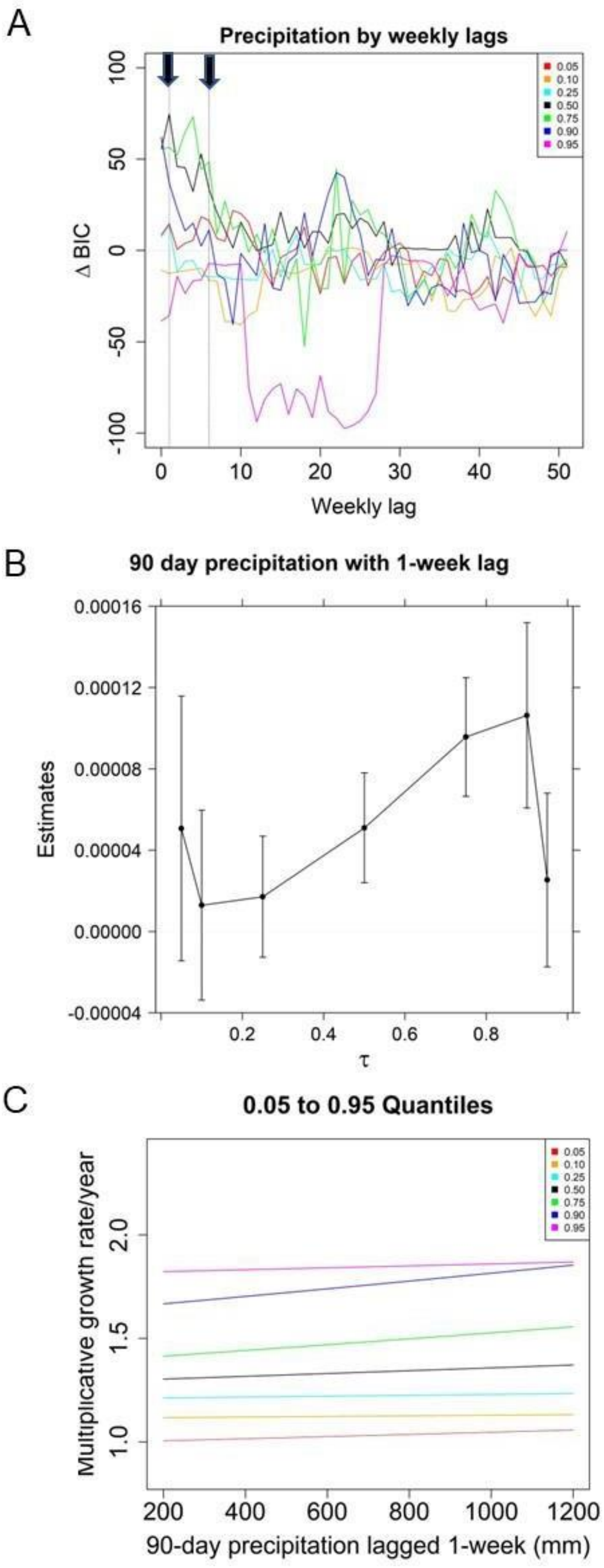
A
Precipitation by weekly lags
Δ BIC
Weekly lag
0.05
0.10
0.25
0.50
0.75
0.90
0.95
B
90 day precipitation with 1-week lag
Estimates
τ
C
0.05 to 0.95 Quantiles
Multiplicative growth rate/year
90-day precipitation lagged 1-week (mm)

**Fig. S5.** (A) Differences in Bayesian Information Criterion (BIC) between the juvenile Brown Treesnake growth rate models that included only the logarithm of prior snout-vent length (SVL) (simple Gompertz growth model with separate intercepts for males and females, with least absolute shrinkage and selection operator (LASSO) shrinkage of intercepts for individual snakes) and models that also included precipitation accumulating over 90 days at different weekly lags. Brown Treesnake data were collected in NW Guam from 10 May 2004 to 18 July 2013. Vertical lines indicate the weekly lagged values that had highest support. (B) Estimated coefficients and 95% confidence intervals for 90- day precipitation lagged 1-week by $\tau$ = {0.05, 0.10, 0.25, 0.50, 0.75, 0.90, 0.95} in the Gompertz model that included separate intercepts for males and females. (C) Model predicted annual growth rates in Brown Treesnakes on Guam as a function of the cumulative rainfall over 90 days and lagged 1 week. This is a Gompertz model where both sexes change similarly with logarithm of prior SVL but sexes have differing intercepts scaled to equal log(700 mm). All models included the LASSO shrinkage of separate intercepts for individual snakes. Shown here are model predictions for females with prior SVL = 700 mm for $\tau$ = {0.05, 0.10, 0.25, 0.50, 0.75, 0.90, 0.95}.

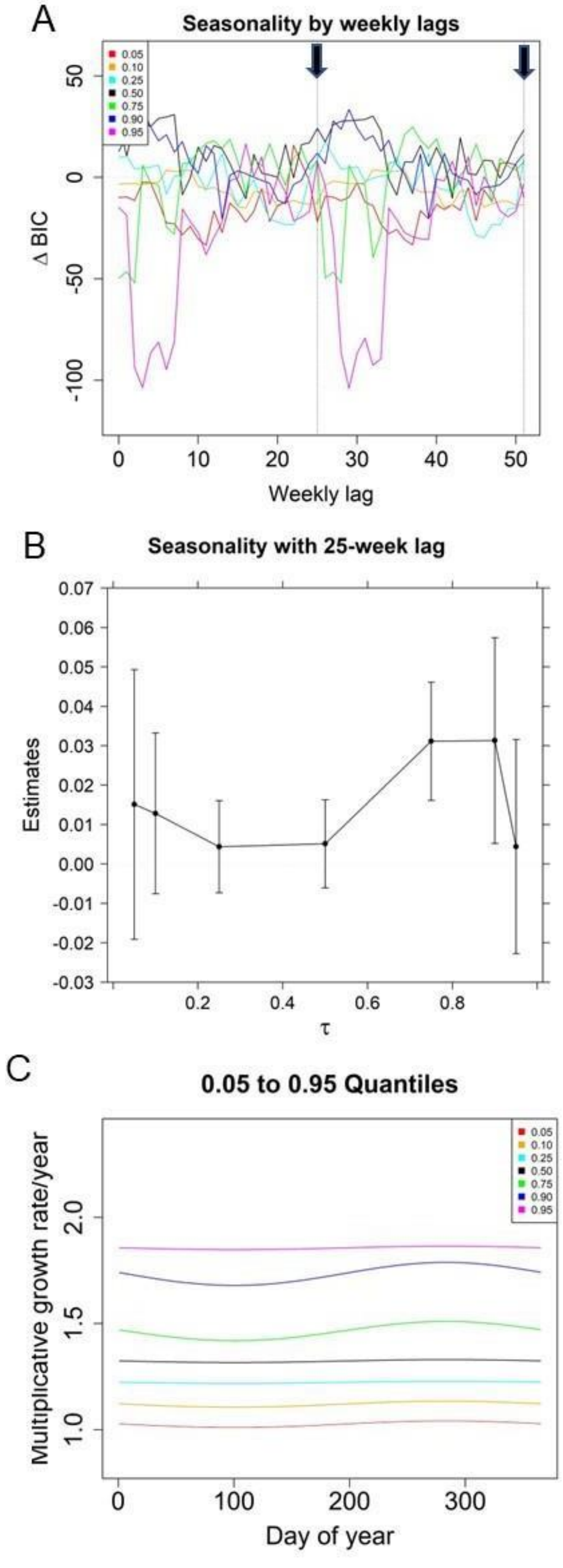
A
Seasonality by weekly lags
0.05
0.10
0.25
0.50
0.75
0.90
0.95
Δ BIC
Weekly lag
B
Seasonality with 25-week lag
Estimates
τ
C
0.05 to 0.95 Quantiles
Multiplicative growth rate/year
Day of year

**Fig. S6.** (A) Differences in Bayesian Information Criterion (BIC) between the Brown Treesnake growth rate models that included only the logarithm of prior snout-vent length (SVL) (simple Gompertz growth model with separate intercepts for males and females, with least absolute shrinkage and selection operator (LASSO) shrinkage of intercepts for individual snakes) and models that also included cyclical seasonality at different weekly lags. Vertical lines indicate the weekly lagged values that had highest support. (B) Estimated coefficients and 95% confidence intervals for seasonality lagged 25-weeks by $\tau$ = {0.05, 0.10, 0.25, 0.50, 0.75, 0.90, 0.95} in the Gompertz model that included separate intercepts for males and females. (C) Model predicted annual growth rates in juvenile Brown Treesnakes on Guam (data were collected from 10 May 2004 to 18 July 2013) as a function of a sinusoidal SEASON covariate with a phase of 365 days and values oscillating between -1 and +1, lagged 25 weeks. This is a Gompertz model where both sexes change similarly with logarithm of prior SVL but sexes have differing intercepts scaled to equal log(700 mm). All models included the LASSO shrinkage of separate intercepts for individual snakes. Shown here are model predictions for females with prior SVL = 700 mm by $\tau$ = {0.05, 0.10, 0.25, 0.50, 0.75, 0.90, 0.95}.

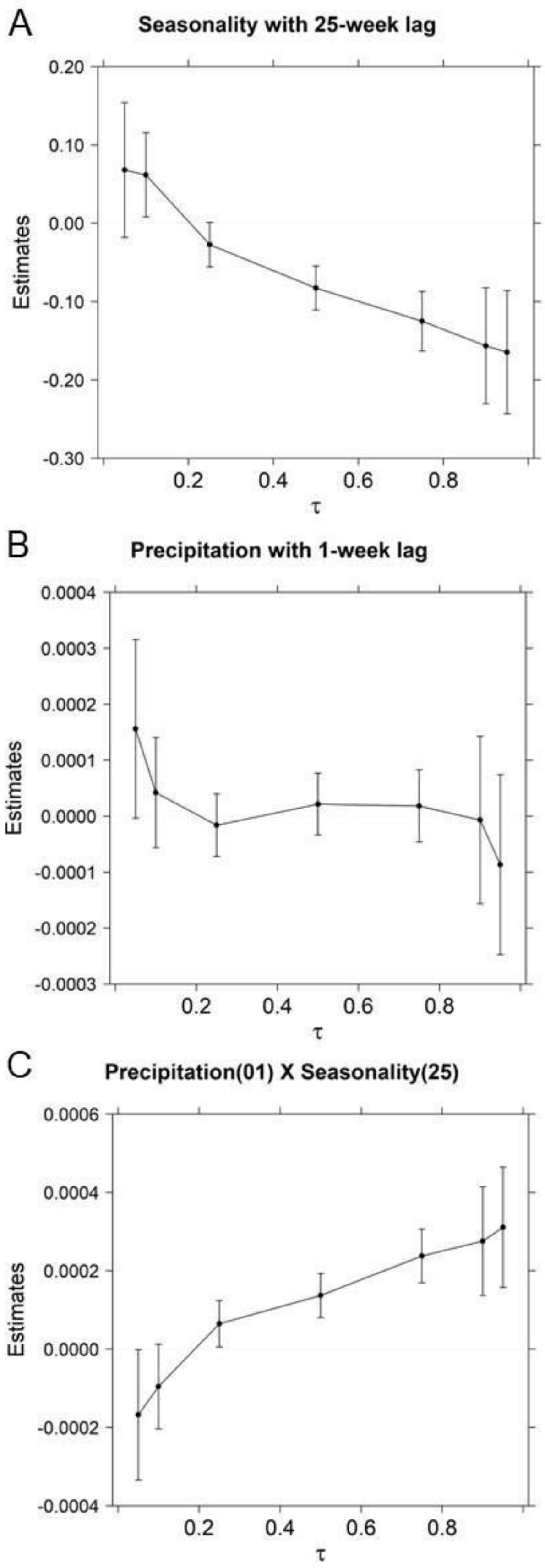
A
Seasonality with 25-week lag
Estimates
0.20
0.10
0.00
-0.10
-0.20
-0.30
0.2
0.4
0.6
0.8
τ
B
Precipitation with 1-week lag
Estimates
0.0004
0.0003
0.0002
0.0001
0.0000
-0.0001
-0.0002
-0.0003
0.2
0.4
0.6
0.8
τ
C
Precipitation(01) X Seasonality(25)
Estimates
0.0006
0.0004
0.0002
0.0000
-0.0002
-0.0004
0.2
0.4
0.6
0.8
τ

**FIG. S7.** Parameter estimates and 95% confidence intervals for linear quantile regression ($\tau$ = {0.05, 0.10, 0.25, 0.50, 0.75, 0.90, 0.95}) models of juvenile male (*n* = 1,517 for 142 individuals) and female (*n* = 1,302 for 128 individuals) Brown Treesnake growth rates in a Gompertz model of logarithm of prior SVL (common slopes for males and females) that includes cyclical seasonality with a 25-week lag (panel A), 90-day cumulative precipitation with a 1-week lag (panel B), and the interaction of these two terms (panel C). Brown Treesnake data were collected in NW Guam from 10 May 2004 to 18 July 2013.

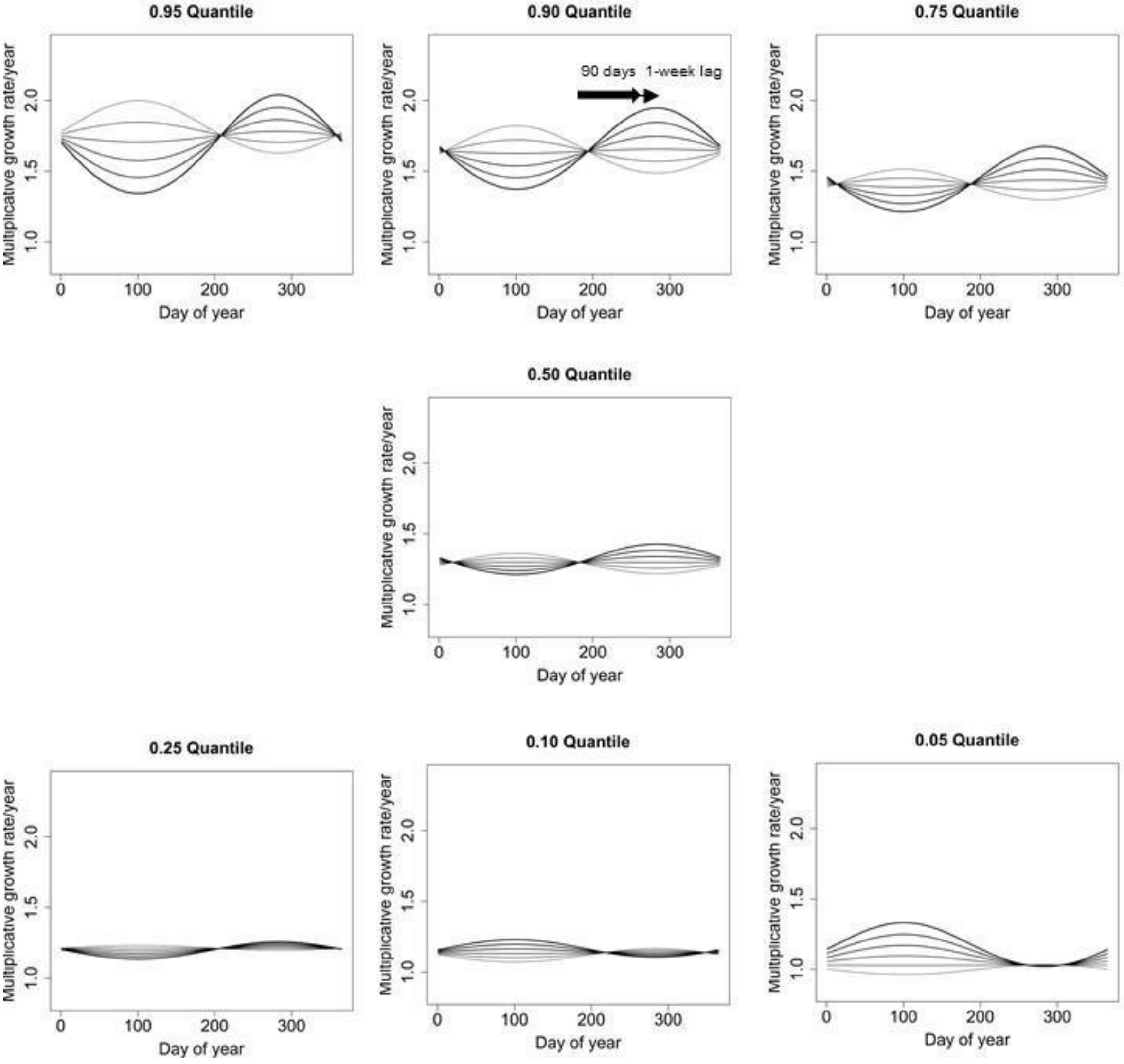


**FIG.S8.** Model predicted annual growth rates for Brown Treesnakes on Guam as a function of the interactive effects of cumulative rainfall and seasonality, when rainfall is measured over 90 days and lagged 1 week and when seasonality is lagged 25 weeks. This is a Gompertz model where both sexes change similarly with logarithm of prior snout-vent length (SVL) but sexes have differing intercepts scaled to equal log (700 mm). All models included the least absolute shrinkage and selection operator (LASSO) shrinkage of separate intercepts for individual snakes. Shown here are model predictions for females with prior SVL = 700 mm. For each of seven growth quantiles (indicated in each panel), model predictions were made for six different amounts of cumulative rainfall, with thicker lines indicating higher values: 200, 400, 600, 800, 1000, and 1200 mm. At other values of SVL, and/or for males, the pattern of changes by day of year and precipitation will be identical by quantiles although the specific values would change. In the upper center panel, the thick black arrow indicates the 90-day time frame over which rain accumulated and thin black line its 1-week lag for the point in time when snakes would be most affected by that past rainfall. Brown Treesnake data were collected in NW Guam from 10 May 2004 to 18 July 2013.
